# MechBERT: Language Models for Extracting Chemical and Property Relationships about Mechanical Stress and Strain

**DOI:** 10.1021/acs.jcim.4c00857

**Published:** 2025-01-31

**Authors:** Pankaj Kumar, Saurabh Kabra, Jacqueline M. Cole

**Affiliations:** † Cavendish Laboratory, Department of Physics, University of Cambridge, J. J. Thomson Avenue, Cambridge CB3 0HE. U.K.; ‡ ISIS Neutron and Muon Source, STFC Rutherford Appleton Laboratory, Harwell Science and Innovation Campus, Didcot OX11 0QX, U.K.; § Research Complex at Harwell, Rutherford Appleton Laboratory, Harwell Science and Innovation Campus, Didcot OX11 0FA, U.K.

## Abstract

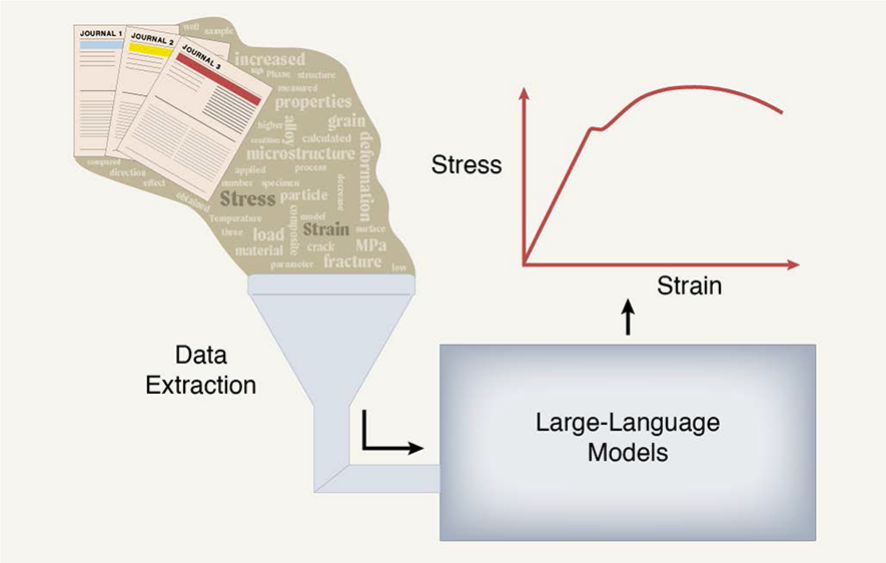

Language models are transforming materials-aware natural-language processing by enabling the extraction of dynamic, context-rich information from unstructured text, thus, moving beyond the limitations of traditional information-extraction methods. Moreover, small language models are on the rise because some of them can perform better than large language models (LLMs) when given domain-specific question-answer tasks, especially about an application area that relies on a highly specialized vernacular, such as materials science. We therefore present a new class of MechBERT language models for understanding mechanical stress and strain in materials. These employ Bidirectional Encoder Representations for transformer (BERT) architectures. We showcase four MechBERT models, all of which were pretrained on a corpus of documents that are textually rich in chemicals and their stress–strain properties and were fine-tuned on question-answering tasks. We evaluated the level of performance of our models on domain-specific as well as general English-language question-answer tasks and also explored the influence of the size and type of BERT architectures on model performance. We find that our MechBERT models outperform BERT-based models of the same size and maintain relevancy better than much larger BERT-based models when tasked with domain-specific question-answering tasks within the stress–strain engineering sector. These small language models also enable much faster processing and require a much smaller fraction of data to pretrain them, affording them greater operational efficiency and energy sustainability than LLMs.

## Introduction

The automatic generation of material databases from the scientific literature has largely followed a step-by-step approach to data extraction. Thus far, information-extraction pipelines have been used to sequentially process an input through many different natural-language-processing (NLP) stages, including tokenization, chemical-named-entity recognition, text parsing, and relation extraction.^1^ With domain-specific adaptations, this approach has demonstrated capability in extracting reliable experimental data at large scales, which are efficiently organized into databases suitable for data-driven research.^2^ To this end, the ‘chemistry-aware’ NLP-based text-mining tool, ChemDataExtractor,^3−6^ has shown particular utility in autogenerating large repositories of experimental data on chemicals and their properties that suit various domains of materials science; see, for example, autogenerated databases of experimental measurements for thermoelectrics,^7^ refractive indices and dielectric constants,^8^ molecules exhibiting thermally activated delayed fluorescence,^9^ semiconductors,^10^ magnetism,^11^ batteries,^12^ photovoltaics,^13^ and photocatalysis for water-splitting applications.^6^

These methods have also been employed in our recent work on stress-engineering materials to curate a repository of related properties,^14^ where grain-size and yield-strength values are associated in the postprocessing stage of database autogeneration and used to statistically validate the Hall–Petch relation^15^ at a much larger scale compared to other work which is all manual.

While these methods directly enable data-driven research, a sequential NLP-based approach to information extraction of experimental data can experience limitations and have significant shortcomings. Methods used for text parsing and relation extraction need to account for all the nuances in the text; a rule-based approach is time-consuming to construct, requires expert knowledge, and has limited adaptability when applied to varied sentences or domains; and machine-learning-based methods require a large amount of manually annotated experimental data for the specific task at hand, which are rarely available. Chemical-named-entity recognition and tokenization processes experience similar challenges, and the sequential nature of conventional NLP-based information extraction means that errors, at any stage of the pipeline, propagate and reduce the overall quality of an eventual knowledge base.

Another practical limitation of conventional NLP-based methods is that they focus on the extraction of independent-property information. Yet, if one is to properly extract material relations accurately with complete information, complex knowledge representations of the target properties and their connection to one another in the text need to be developed which is an exceedingly difficult task as is often reflected in the final precision and recall metrics of autogenerated databases. For example, Isazawa and Cole implemented a nested knowledge representation for the extraction of photocatalysis data; in which, the F-score for the primary property remained high, but nested relations containing experimental conditions exhibited much lower F-scores, dipping to around 15% in some cases.^6^ When designing a knowledge representation for stress–strain properties, not only do factors related to manufacturing, processing, experimental, and measuring conditions need to be considered; but also, the semantic interrelations between mechanical properties, such as yield strength, tensile strength, and Young’s modulus, which are equally complex and diverse. A lack of such knowledge representations can lead to false positives in the extracted data, especially when multiple properties are discussed within the same text; accurately associating extracted values with the correct property being referenced becomes increasingly challenging. Therefore, the task of conceptualizing and programming a complete knowledge representation for properties found on the stress–strain curve is crucial, yet it is nontrivial, and its inherent difficulty will be evident in the final precision and recall metrics of a resulting stress-engineering database.

Fortunately, the field of NLP, and in particular the statistical modeling of language, has been rapidly progressing. Recent research has focused on large language models that are mostly built on the Transformer architecture,^16^ such as Bidirectional Encoder Representations from Transformers (BERT),^17^ the Generative Pretrained Transformer (GPT) series of language models,^18,19^ the Robustly optimized BERT pretraining approach (RoBERTa),^20^ and the Text-to-Text Transfer Transformer (T5).^21^ These have all demonstrated state-of-the-art performance on a variety of natural-language tasks, from question-answering to text generation. BERT-based models and others tackle natural-language tasks in two stages. First, the model is pretrained on a large set of unlabeled text to learn the general-purpose language representations, allowing the model to understand the context and meaning of language, even when supplied with unseen sentences. The pretrained model is then fine-tuned for a specific task using labeled data, allowing the embedded knowledge to be repurposed for different tasks depending on the use case.

In the context of information extraction from the scientific literature, a pretrained language model can be fine-tuned for question-answering tasks where the embedded knowledge can be utilized for extractive purposes. This approach alleviates many of the aforementioned problems that are encountered with the conventional NLP-based information-extraction pipeline. In particular, stages such as tokenization, named entity recognition, and text parsing are learnt simultaneously when pretraining a language model, thereby precluding the need for individual methods or expertly crafted rules to be developed for each; furthermore, the semantic connection between properties is automatically learned from the unlabeled text during the pretraining process, removing the need to manually design complex knowledge representations and enabling more accurate extraction of material properties from the text. Thus, language models have the potential to address some of the shortcomings of sequential NLP extraction pipelines.

However, general-purpose language models, such as BERT, may struggle to capture domain-specific characteristics. As such, specialized BERT models, tailored to the unique linguistic patterns of a particular field, have been developed and demonstrate superior performance on downstream, domain-specific, tasks. For instance, BioBERT,^22^ FinBERT,^23^ SciBERT,^24^ and ChemBERTa^25^ (or even CamemBERT^26^ for the French language) have all been pretrained on text from their respective fields and achieved high performance in later natural-language tasks. Notably, BatteryBERT^27^ and OpticalBERT^28^ have surpassed other BERT-based models and conventional information-extraction pipelines. In contrast to other domains, no such model has been reported that is specialized for stress–strain information. Therefore, it is natural to question whether a similar type of BERT model could be useful for information extraction of stress–strain-related properties.

To this end, four BERT models are developed in this study using different pretraining corpora, with a focus on text that contains stress–strain information, which is sourced from the scientific literature. Due to the nature of the corpora, including a plethora of mechanical properties, the four models are aptly named as a class of *MechBERT* models. Once parameters have been optimized for fine-tuning, it will be demonstrated that the domain-specific models perform better than other BERT-based models for extractive tasks within the field of stress engineering while also maintaining performance on general English-language question-answering data sets.

For evaluation, a domain-specific question-answering data set was curated with the help of an annotation tool that has been purpose built in this work. The results herein will cement the importance of corpus specificity in pretraining language models for improved performance on downstream tasks within that domain; with MechBERT models outperforming language models that are not only pretrained on many more data but which are also significantly larger in terms of their number of model parameters. As such, our MechBERT models have the potential to be integrated into information extraction systems to overcome the limitations of sequential NLP extraction methods.

**Figure 1:**
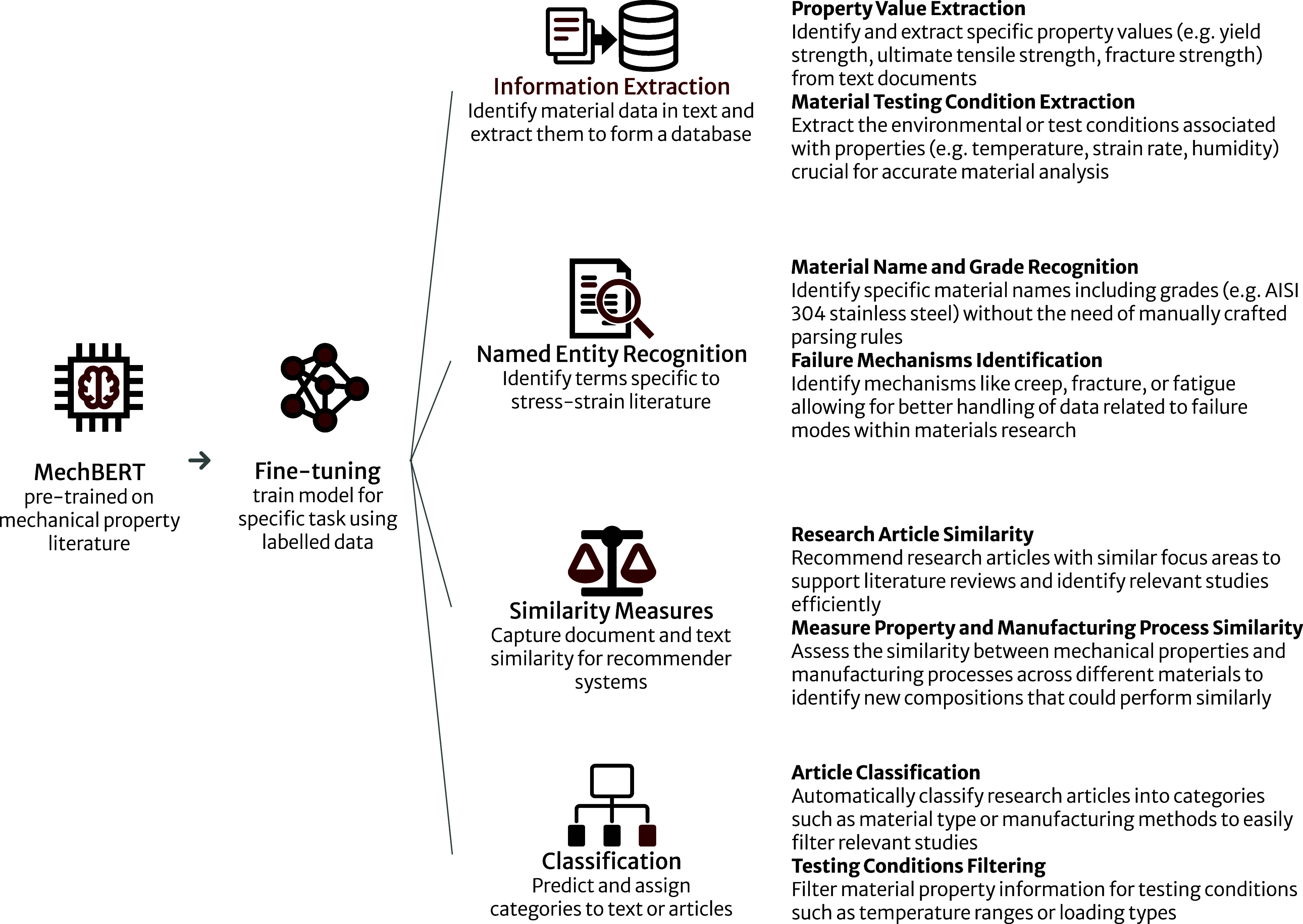
Examples of a few different use cases of our MechBERT models. In this study, we fine-tune MechBERT for extractive question-answering, which helps facilitate information extraction. Other researchers may find successful application of our MechBERT models in a variety of NLP tasks, including named entity recognition, similarity measurement, and classification tasks.

While this paper focuses on the development of MechBERT models and assessment of their effectiveness for stress–strain engineering information-extraction tasks, it is worth highlighting the broader applicability and importance that these material-domain-specific MechBERT models have for the mechanical engineering community. Our MechBERT models provide a foundation that can be fine-tuned to enhance performance across a wide range of downstream tasks tailored to the specific needs of this field. Figure 1 illustrates example use cases in which a stress–strain engineering language model, further optimized through fine-tuning, could be applied. Therefore, our overarching development of domain-specific MechBERT models offers a vehicle for the materials-engineering research community to interact with carefully crafted language models about the mechanical properties of chemicals and tailor them for their bespoke needs.

## Methods

To explore the benefits of domain-specific language models for information extraction of properties found on the stress–strain curve, four models were developed in this study: PureMechBERT-cased, PureMechBERT-uncased, MechBERT-cased, and MechBERT-uncased. These models were fine-tuned for question-answering tasks, as this enables the embedded knowledge to be used for extractive tasks. This section will detail the pretraining and fine-tuning processes of these language models alongside training-data preparation and a tool that we have built for creating question-answering data sets.

### Model Overview

The model architecture follows the original model that was discussed by Devlin et al.^17^ to ensure consistency and allow for fair comparison. Pretraining model parameters are chosen such that the total number of parameters matches that of the original BERT models. The HuggingFace implementation of BERT, found in their *Transformer* package,^29^ was used, and the relevant parameters are listed in Table 1. Each BERT model has a total of 110 million parameters, and the four BERT models were pretrained on different corpora; PureMechBERT models were pretrained on only the domain-specific corpus, while MechBERT models were further pretrained starting from the weights of the original BERT_BASE_ model.

**Table 1:** Summary of the Main Configuration Parameters That Define the Architecture of the BERT Model

parameter	value
vocabulary size	30,522
hidden size	768
hidden layers	12
attention heads	12
dropout probability	0.1
transformer version	4.25.1

Alternative model architectures were also considered for our domain-specific language models, including certain increasingly popular Generative Pretrained Transformer (GPT) language models.^18,19^ However, many downstream domain-specific tasks, such as extractive question answering or chemical entity recognition, require precise, factual answers. In such cases, other transformer-based language model architectures can be more suitable, as GPT models tend to be quite susceptible to producing hallucinations, or they can necessitate additional postprocessing to ensure accuracy. Therefore, we utilize the BERT architecture in this study. Nonetheless, due to the widespread popularity of tools such as ChatGPT, further discussion and comparison between ChatGPT and our MechBERT models are provided in the Supporting Information of this paper.

### Pretraining Corpus

In this study, the pretraining corpus is composed of scientific articles that have been published by Elsevier, Springer Nature, and Wiley. For the former two publishers, the previously cultivated corpus by Kumar et al.^14^ is further extended to contain more general stress–strain information through the use of additional search queries for relevant mechanical properties, following the method discussed therein. The text from each Elsevier and Springer Nature article was extracted using the reader package of ChemDataExtractor^3^ and then combined into a single text file.

Unique to this study, access to the Wiley API was granted and was integrated into the article retrieval pipeline. However, this API is limited and does not provide an interface to search the Wiley article repositories; as such, the DOI of relevant articles had to be searched for separately. While other APIs, such as CrossRefAPI,^30^ could be used for the search process, it was found that articles not relevant to the search query would often be returned via such an approach, which would negatively skew pretraining toward irrelevant text. Instead, the online Wiley search engine was used directly to gather a list of relevant DOIs; although, the number of relevant articles identified comprised less than 1% of the overall pretraining corpus.

**Figure 2:**
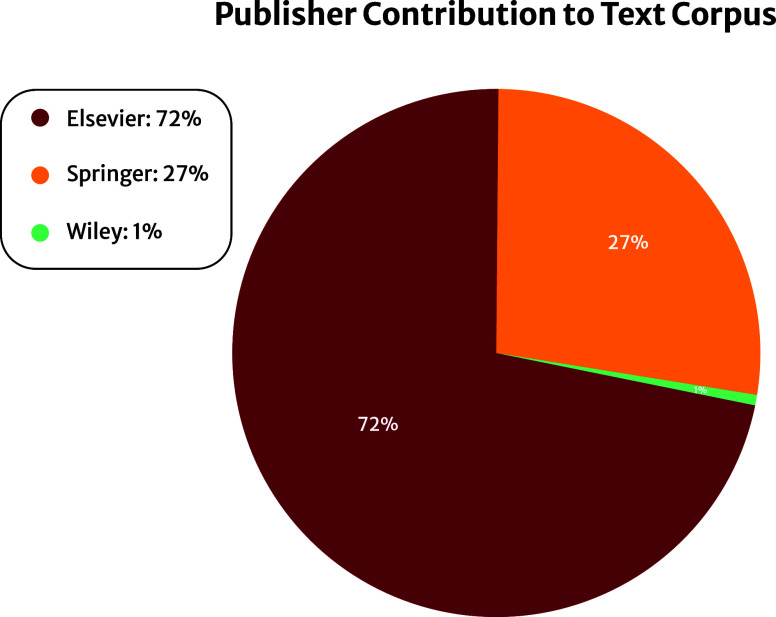
Percentage contribution of each publisher to the domain-specific text corpus curated in this study.

Additionally, the Wiley API provides full-text articles only as Portable Document Format (PDF) files, which often causes difficulties in retrieving the contained text. Nonetheless, the plain text of these PDF files was extracted using PDFDataExtractor^31^ and consolidated into a single text file, as the inclusion of any additional text data is useful for pretraining each language model. Overall, the domain-specific text corpus consists of approximately 1.2 billion tokens sourced from over 400,000 articles, and the relative contribution of papers from each publisher is shown in Figure 2. In contrast, the original BERT_BASE_ model was pretrained on a corpus of 3.3 billion tokens that had been sourced from English Wikipedia and the Book Corpus.^32^ While the domain-specific corpus used in this study is smaller than this corpus of generic English text, it is still within the range of optimal parameter/training token allocation.^33^

**Figure 3:**
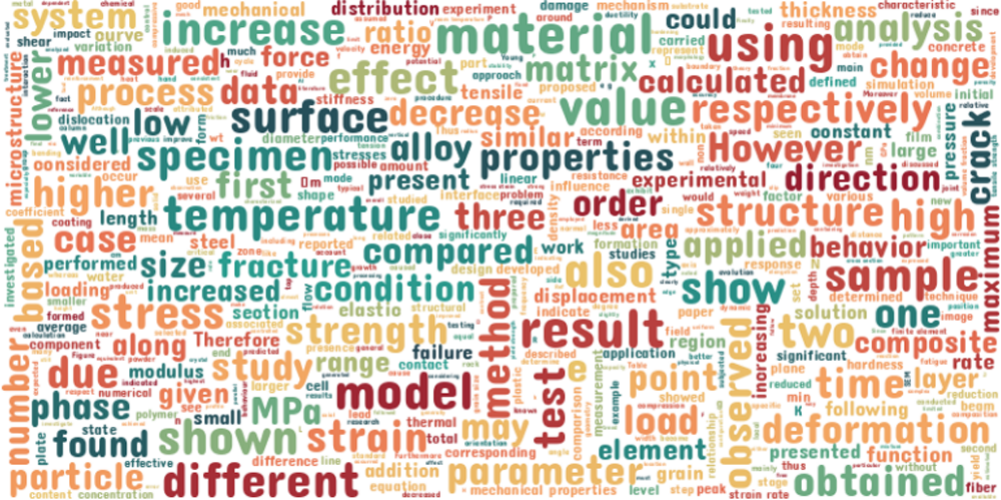
Word cloud of the most frequent words contained within our domain-specific corpus.

A word-cloud visualization was generated to illustrate the vocabulary distribution of our domain-specific corpus. As shown in Figure 3, high-frequency terms are emphasized, including “stress”, “fracture”, “tensile”, “strength”, and even the commonly used units for tensile properties, “MPa”. These further highlight the uniqueness of the collected text compared to other general purpose corpora, enabling the language model to learn the distinct linguistic patterns that are prevalent in the scientific literature, especially those concerning stress–strain property information.

### Tokenization

To prepare the corpus for pretraining the language models, the text was tokenized using WordPiece embeddings,^34^ following the approach used by the original BERT model.^17^ These are subword representations that allow for the tokenization of a corpus using a smaller vocabulary; thus, they are more computationally efficient.

For the MechBERT models, the original cased and uncased BERT WordPiece tokenizers were used, whereas new tokenizers were trained for the PureMechBERT models. The training made use of the HuggingFace libraries^29^ and the domain-specific corpus to generate a vocabulary of the same size for both cased and uncased variants. The entire corpus was tokenized using the respective WordPiece tokenizers of each model to convert the text into appropriate input embeddings for the BERT architecture.^17^

**Figure 4:**
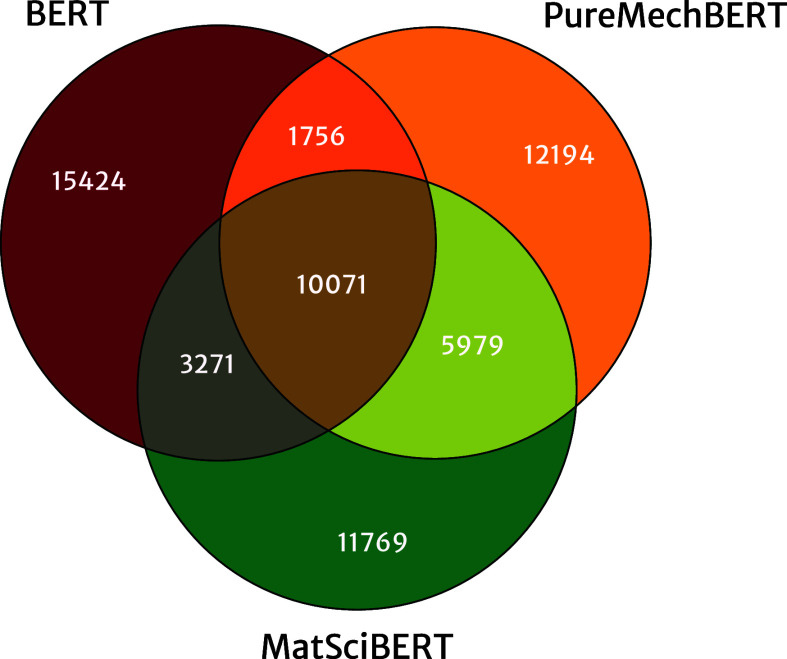
Overlap of the different vocabularies used for BERT,^17^ MatSciBERT,^35^ and PureMechBERT models.

As the vocabularies were obtained directly from the corresponding corpus, they are valuable tools for the comparison of different training sets used in distinct language models. The vocabulary overlap between three distinct language models is shown in Figure 4. This highlights the level of uniqueness between PureMechBERT, BERT,^17^ and MatSciBERT,^35^ the latter being a BERT model that was trained on a corpus that encompasses all of material science. The MatSciBERT model was specifically chosen for comparison, as its material-science-focused corpus includes alloys and ceramics, which are often the target of stress–strain studies. Consequently, the PureMechBERT vocabulary has a greater overlap with MatSciBERT than with BERT, with 53 and 39% overlapping word pieces, respectively. Qualitatively, the vocabularies of PureMechBERT and MatSciBERT models are more similar than they are to those of BERT owing to the scientific nature of their corpora. Still, their significant differences from the foundational language model suggest that specialized terms that are unique to the scientific corpora are prevalent. Alongside the word cloud in Figure 3, Figure 4 demonstrates the focus of the corpus toward stress–strain information, which in turn will be reflected in the pretrained language model.

### Pretraining

Following text processing and tokenization, the next stage involves pretraining the four language models: cased and uncased variants of both PureMechBERT and MechBERT. The PureMechBERT models were initialized from scratch and were trained solely on the stress–strain corpus that was curated in this work. In contrast, MechBERT models were initialized from the pretrained weights of the original cased and uncased BERT_BASE_ models. This was followed by further training on the domain-specific corpus, meaning that knowledge from both general and scientific texts is embedded into those models.

During the pretraining stage, the masked language modeling objective was used for all models. This involves masking 15% of tokens within the pretraining corpus at random and requires the model to correctly predict the masked words. The HuggingFace Transformers library^29^ provided the implementation for this task. The original BERT paper also employed next-sentence prediction for pretraining;^17^ however, Liu et al. demonstrated that this does not correspond to a significant difference in the overall performance.^20^ As such, this task was removed from the pretraining objective in this study to reduce the overall computational cost.

Pretraining was performed using NVIDIA A100 GPUs within the Polaris cluster at the Argonne Leadership Computing Facility (ALCF), Illinois, USA. A total of ten computing nodes were used, each containing four GPUs. Pretraining took between 15 and 20 h to complete for each variant. DeepSpeed^36^ was employed to distribute training across multiple nodes, resulting in more efficient processing and a reduction in the time taken for pretraining. Moreover, a critical factor in pretraining is the cumulative number of training sequences that are processed. The original BERT model and other domain-specific adaptations, such as BatteryBERT^27^ and OpticalBERT,^28^ pretrained their models for 1 million steps with a batch size of 512, resulting in the processing of 2.56 × 10^8^ sequences in total.^17^ In this work, pretraining was optimized while maintaining the overall exposure to training sequences. To this end, a total batch size of 2048 was used, and the training steps were reduced to 125,000 and 187,500 for MechBERT and PureMechBERT models, respectively. This approach maintained model performance while reducing the total pretraining time. The increased exposure to training sequences for the PureMechBERT models accommodated their initialization from scratch. This also aligned with BatteryBERT models that were trained from scratch, which were trained for a total of 1.5 million steps with a batch size of 256.^27^

### Fine-Tuning for Question Answering

During the fine-tuning stage of language-model constructions, the pretrained model architecture was kept largely the same with the learned weights being “frozen”. Only the parameters associated with task-specific input and output layers were adjusted using labeled data. For information extraction, the models were fine-tuned for the question-answering tasks. This involved adding a single output layer, often referred to as the “answer head”, as suggested in the original BERT paper.^17^ This layer receives questions and context pairs that have been encoded by the pretrained BERT model. The answer head predicts the location of an answer span within the context by identifying the token positions *i* and *j* (where *i* ≤ *j*) that exhibit the highest dot product scores with learned start and end vectors, i.e., maximizing *S*·*T*_*i*_ + *E*·*T*_*j*_ for *j* ≥ *i*, where *T*_*i*_ is the final hidden vector for the *i*^th^ input token, and *S* and *E* are the start and end vectors, respectively. By comparing these predicted answer locations with the “ground-truth” labels provided in an annotated training data set, solely the parameters of the new answer head need to be optimized, meaning that a minimal number of parameters need to be learnt from scratch. This approach allows the model to use the knowledge learnt during pretraining without additional extensive training. Indeed, fine-tuning is much less computationally expensive than pretraining and can be completed within a few GPU node hours.

All models were fine-tuned on the Stanford Question and Answering Data set (SQuAD) v2.^37^ This data set is used as a benchmark for question-answering systems and contains 150,000 question-context pairs that have been annotated with crowd-sourced answers. Of these, 50,000 questions are unanswerable and have been labeled as such; this is in contrast to its predecessor, SQuAD v1,^38^ which only includes answerable question-context pairs and has been employed in related studies, e.g., by Huang et al. in BatteryBERT^27^ and Zhao et al. in OpticalBERT.^28^ However, in real-world applications, information-extraction systems will frequently encounter passages of text that miss the target information. A suitable model must not only correctly find an answer span within the context but also identify instances where no answer exists. Therefore, our models were primarily fine-tuned on SQuAD version 2.0 for information extraction purposes. To allow for comparison with other studies that used SQuAD v1, an additional set of fine-tuned models on this data set was produced. Fine-tuning was carried out using the training scripts provided by HuggingFace.^29^

Determining the best hyperparameters for fine-tuning a model can be a time-consuming and inefficient process, particularly when using a random or grid-search manual approach, which can easily overlook the best set of parameters. To this end, the computationally inexpensive nature of fine-tuning was leveraged to find the optimal set of hyperparameters by using Bayesian optimization to guide the search. The search space of parameters used in this study is listed in Table 2, with the goal of maximizing the exact-match evaluation metric which reflects the percentage of correctly answered questions. This was facilitated by the Weights & Biases (W&B) framework.^39^

**Table 2:** Search Space for Finding the Optimal Hyperparameters for Fine-Tuning of BERT-Based Models

hyperparameter	min	max
learning rate	1 × 10^–8^	1 × 10^–4^
number of epochs	1	20
batch size	4	256

An initial set of hyperparameter configurations was chosen at random to gather sample data to construct a surrogate model. In the W&B framework, this utilizes a Gaussian process regression model that captures the relationship between hyperparameter selection and the exact match score. Using the surrogate model and expected improvement, hyperparameters are suggested that balance ones that are expected to perform well and the ones that explore new regions of the hyperparameter space. Iteratively, models are fine-tuned using the newly suggested configurations, and the resulting exact match score is used to update the surrogate model. This continually improves its ability to estimate performance given a set of hyperparameters, and therefore, it becomes more capable of guiding the search toward the most optimal set that maximizes the exact match score. After approximately 100 iterations, the “best” configuration that achieved the highest exact match score was selected for the final models.

### Domain-Specific Question-Answering Evaluation Data

**Figure 5:**
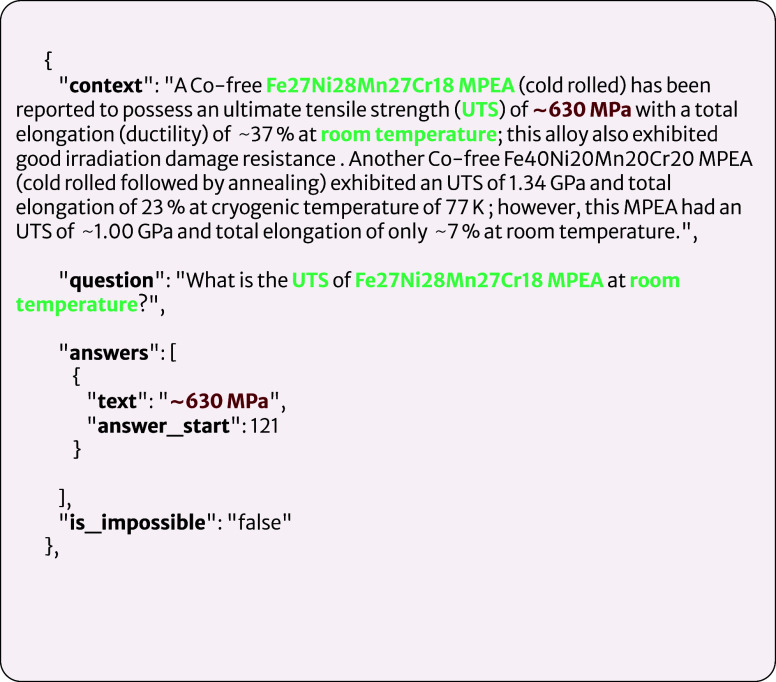
Example SQuAD-like entry in our domain-specific evaluation set.

To evaluate the performance of our language models on domain-specific question-answering tasks, a custom data set was created, following the format of the SQuAD data sets. A total of 411 questions and answers were manually generated, with context paragraphs sourced from articles that were not in the pretraining corpus. Of these, 65 questions are unanswerable and were labeled as such. A sample entry from the evaluation set is shown in Figure 5 and consists of a question-context pair with the corresponding ground-truth answer. The *“answer_start”* field specifies the starting-character index of the answer, allowing for easy conversion to a starting-token index, irrespective of the tokenizer choice, to enable more versatility when assessing different models. This field is also useful in cases where the initial token of an answer appears multiple times in a sequence, as it allows for the answer span to be accurately identified. To account for unanswerable questions, a Boolean field named *“is_impossible”* was included; if false, the answer resides within the context, and the model should predict the answer span; if true, the question does not have a corresponding answer in the given context. The questions within our data set have been written to primarily focus on the extraction of material-property information. As such, an underlying understanding of the knowledge representations between properties within the context is required for accurate answers to be found.

**Figure 6:**
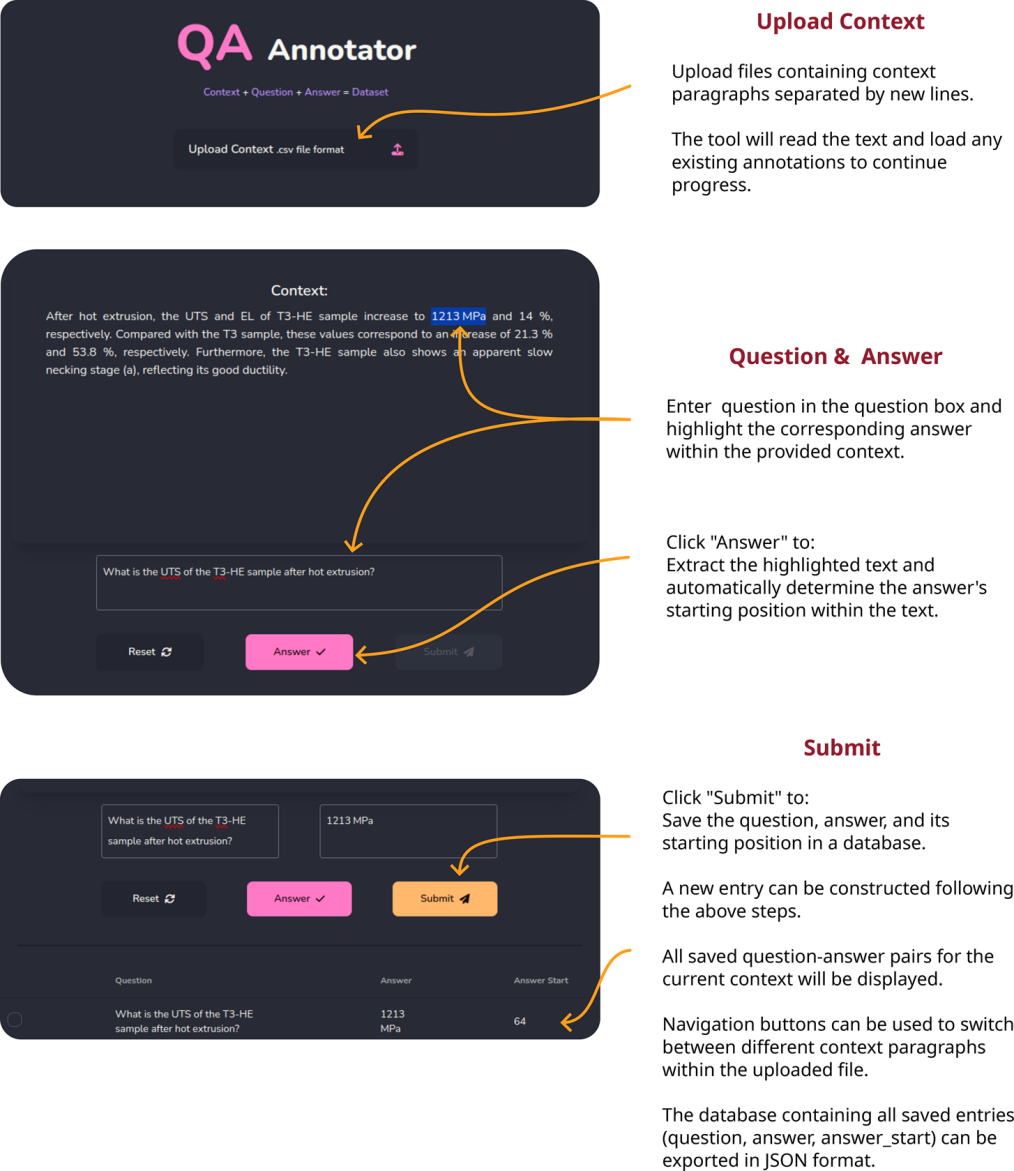
Example usage of the QA Annotator Web Tool that was created for this work.

Approaching the task of creating such data sets without any tool assistance can be tedious and prone to error. Manually typing each field and its contents leads to a higher risk of mislabeling, which does not allow for fair evaluation. Therefore, to facilitate the creation of SQuAD-like data sets, a web tool was designed to streamline the process of annotating context with questions and answers. On the frontend, the JavaScript library for handling web interfaces, ReactJS,^40^ was implemented. The backend was written in Python and utilizes the Django framework.^41^ The interface and annotation steps are exemplified in Figure 6, and the web app has been made openly available on GitHub.^42^

## Results

**Figure 7:**
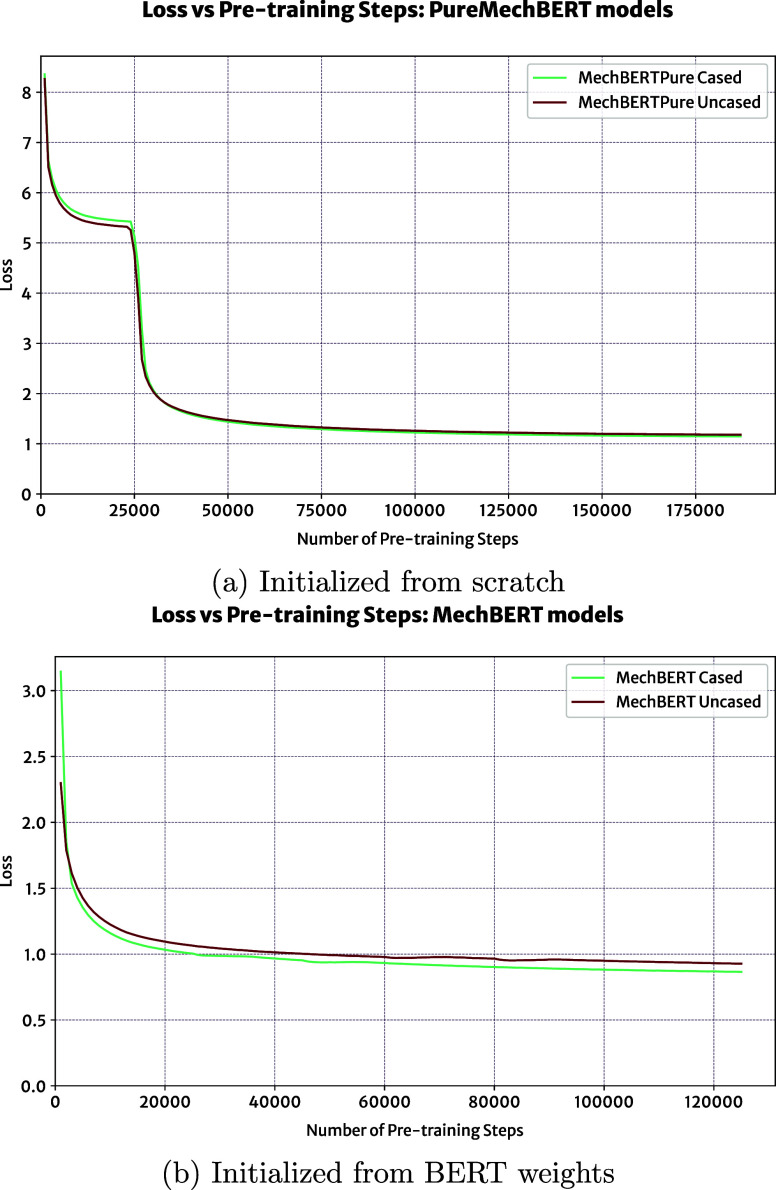
Evolution of loss during pretraining of each model.

The pretraining progress of each model is shown in Figure 7, which depicts the evolution of the loss value, a unitless measure of the relative error between the prediction of masked tokens made by the model and their actual value, over the number of pretraining steps. While not mathematically conclusive, a decreasing loss suggests that the models are continuously learning and improving during the pretraining stage. For all models, the loss curves trend downward and level off without completely stagnating, indicating that the number of pretraining steps is sufficient and balances learning while preventing overfitting. Interestingly, in Figure 7a, the loss curves for the PureMechBERT models initially begin to converge at a loss value of around 5.5. This indicates an initial difficulty in accurately performing the masked language modeling task, and thus, a difficulty in successfully learning the linguistic patterns within the domain-specific corpus, perhaps due to these models being initialized from scratch. However, after around 25,000 pretraining steps, these models overcome a local minima and proceed to converge to a loss value of around 1, which aligns with the loss of MechBERT models and similar BERT models that have been developed for the scientific domain.^27,28^ The loss values of cased BERT models are all lower than their uncased counterparts, highlighting that the case of tokens provides valuable information for predicting masked tokens, and this may be reflected in the performance of downstream tasks. For a more comprehensive evaluation, the relative performance of BERT models fine-tuned for question-answering tasks was evaluated.

### Fine-Tuning Optimization

The optimal hyperparameter configuration for fine-tuning each BERT model is detailed in Table 3. During the search, over 100 different configurations were tested for each model, with each iteration aiming to improve the exact match score on the SQuAD v1 and v2 data sets. This exploration revealed the significant impact of hyperparameter selection; across all models, the average standard deviation of the exact-match score was 6.1%, with highs of 18.4%. In other words, for a given data set and pretrained model, an information-extraction system built with carefully selected hyperparameters could achieve almost 20% fewer errors compared to a suboptimally configured system.

**Table 3:** Optimal Hyperparameter Configurations for Fine-Tuning on SQuAD v1 and v2

dataset	model	learning rate	epochs	batch size
SQuAD v1	MechBERT Cased	5.75409064017 × 10^–5^	3	60
	MechBERT Uncased	6.69796562345 × 10^–5^	3	44
	PureMechBERT Cased	2.34149250752 × 10^–5^	4	24
	PureMechBERT Uncased	6.65858503326 × 10^–5^	3	140
SQuAD v2	MechBERT Cased	6.40600319349 × 10^–5^	2	128
	MechBERT Uncased	1.494572498569 × 10^–4^	2	23
	PureMechBERT Cased	4.90973747641 × 10^–5^	3	76
	PureMechBERT Uncased	3.80820446232 × 10^–5^	4	60

**Figure 8:**
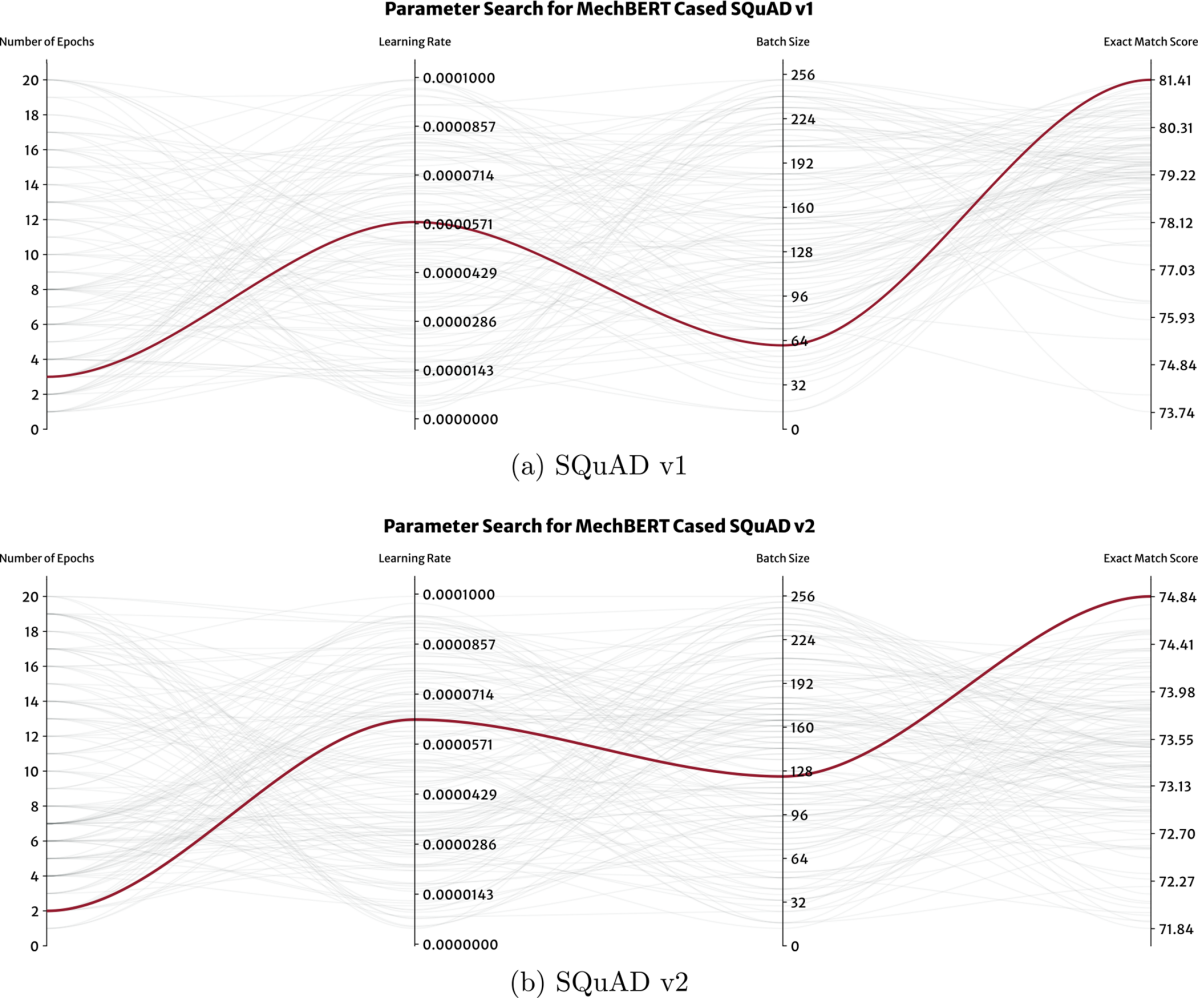
Summary of the top 100 hyperparameters found during the optimization of fine-tuned MechBERT cased models. All configurations are compared using the exact-match score and the best performing model is highlighted in red.

The variability in exact-match scores across different fine-tuning configurations is further illustrated in Figure 8; this showcases the top 100 performing hyperparameter configurations for fine-tuning MechBERT cased models on SQuAD v1 and v2, with the best performing model parameters being highlighted in red. Similar figures for each model can be found in the Supporting Information. These further highlight the benefit of the extensive hyperparameter search performed herein. In particular, they demonstrate the advantage of Bayesian optimization over other methods, such as grid searching, where the optimal configuration may lie outside the predefined value ranges. For instance, grid-search approaches typically test a smaller set of parameter values, such as batch sizes of 16 and 32; learning rates of 5 × 10^–5^, 3 × 10^–5^, and 2 × 10^–5^; or number of epochs ranging from 1 to 4.^27,28^ If a similar method was employed in this study, the resulting performance of the fine-tuned models would be very different, and the best model configuration would be overlooked. In contrast, Bayesian optimization explores the hyperparameter space more efficiently and leads to the identification of configurations that significantly improve the model performance.

With these optimized hyperparameters, our eight fine-tuned models were evaluated for question-answering tasks to demonstrate their capability for use in information extraction. This evaluation encompassed both general-language and domain-specific tasks, using both SQuAD and a data set custom-built for this study. For comparison, the best performing BERT models that have been fine-tuned in related studies of material properties are sourced and tested against the same criteria.

### Evaluating Question-Answering Performance

Performance was evaluated by tasking each model to identify the relevant answer to a given question within a context paragraph. These are provided in an evaluation data set that also contains the “ground-truth” answer to the question. If the predicted answer is identical to the ground-truth in its entirety, then it is considered to be correct and is assigned a score of 1. Conversely, if any part of the prediction deviates from the ground-truth, it is considered to be incorrect and assigned a score of 0. This is analogous to labeling the predicted answer as a “True Positive” or “False Positive” depending on its correctness. The exact-match score of a model is determined by calculating the percentage of predictions that have been correctly made. However, since an answer is correct or not, this metric does not consider answers that are mostly correct. For instance, the question “*What is the Ultimate Tensile Strength (UTS) of Material X?*” given a sentence “*Material X has an UTS of 500 MPa*” can be answered as either “*500 MPa*” or “*UTS of 500 MPa*” which are both correct, but only the one matching the ground-truth would be accepted, resulting in a potential false-positive labeling. To account for this, the general-language SQuAD data set owners crowd sourced their ground-truth answers, and as such, these data sets have multiple variations of answers to a single question. This allows the predictions to be compared to a greater variety of human-annotated answers, minimizing the possibility of factually correct predictions being disregarded. A byproduct of this approach is that the human-level performance can be deduced by comparing the crowd-sourced answers to each other; this demonstrated that humans performing question-answering tasks achieve an exact-match score ranging from 77 to 86% with little difference being observed between the SQuAD data set versions.

The domain-specific data set does not contain multiple variations of answers, as it was constructed using annotations from a single person (the first author of this paper). Instead, the F1 score can be used as a more reliable evaluation metric where each individual token in the prediction is compared to the ground-truth answer to calculate precision and recall. The definitions of precision, recall, and F1 score follow closely to the ones used in related papers.^27,28^ In this case, precision describes the percentage of predicted tokens that are in the ground-truth and are correct, i.e., the accuracy, and recall captures the completeness of predictions by measuring the percentage of ground-truth tokens that appear in the predicted answer. As usual, the F1 score balances both precision and recall to determine the ability of the model to give accurate and complete answers and allows for evaluation that is not strictly “all-or-nothing”, unlike the exact-match score. These metrics are calculated using the following:
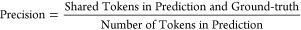
1
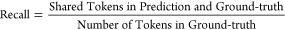
2
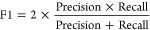
3

#### Evaluating General Text Question-Answering Performance

Summaries of the evaluation metrics on the general English-language data sets, SQuAD v1 and v2, are listed in Tables 4 and 5, respectively. Interestingly, all cased models in this study outperform their uncased counterparts, reflecting the trend in the loss function observed in their pretraining. This stands to reason since the cased variants have been provided with additional information such as the capitalization of proper nouns to help perform the masked-language modeling task, which also seems to carry over for question-answering tasks. Both cased and uncased versions of the MechBERT models maintain a similar level of performance to the BERT_BASE_ model, with the cased version performing marginally better when dealing with only answerable questions (SQuAD v1). At the same time, MechBERT models perform slightly better than the BERT_BASE_ models with the inclusion of unanswerable questions (SQuAD v2). This result may be due to the more optimized approach used to fine-tune the MechBERT models. Despite the pretraining corpus in this study being largely focused on scientific texts that are “out-of-scope” for the general English language question-answering task at hand, the further pretraining of MechBERT from the original BERT weights does not negatively impact the performance of our models on downstream general question-answering tasks.

**Table 4:** Model Performance on SQuAD v1

model	exact-match (%)	F1 score (%)
MechBERT Cased	81.41	88.61
MechBERT Uncased	80.41	87.95
PureMechBERT Cased	76.81	84.79
PureMechBERT Uncased	75.18	83.85
BERT_BASE_^17^	80.80	88.50
BatteryOnlyBERT Cased^27^	79.61	87.30
BatteryOnlyBERT Uncased^27^	79.53	87.22
MatSciBERT^a^^35^	77.42	85.73

a The MatSciBERT model was fine-tuned following the guidelines of the BERT_BASE_ model.

**Table 5:** Model Performance on SQuAD v2

model	exact-match (%)	F1 score (%)
MechBERT Cased	74.84	77.95
MechBERT Uncased	74.78	77.89
PureMechBERT Cased	71.77	74.84
PureMechBERT Uncased	71.06	74.52
BERT_BASE_ Cased^17,43^	71.15	74.67
BERT_BASE_ Uncased^17,43^	73.68	77.88
MatSciBERT^a^^35^	73.14	76.46

a The MatSciBERT model was fine-tuned following the guidelines of the BERT_BASE_ model.

However, PureMechBERT models perform worse than MechBERT models since they were pretrained from scratch on purely the domain-specific corpus, which only shares 39% of the original BERT vocabulary. Thus, the general English-language knowledge that was already embedded into the original BERT weights is not present in PureMechBERT models, meaning that PureMechBERT models are not suited for downstream tasks that are primarily focused on general language. Moreover, the corpus used to pretrained PureMechBERT models is relatively small, meaning that these pretrained models were not supplied with enough samples of general English-language patterns to properly tackle such tasks. In comparison, BatteryBERT pretrains models from scratch (BatteryOnlyBERT) using a corpus that is almost 40% larger than those of PureMechBERT models;^27^ hence, performing better on question-answering tasks from SQuAD v1.

**Figure 9:**
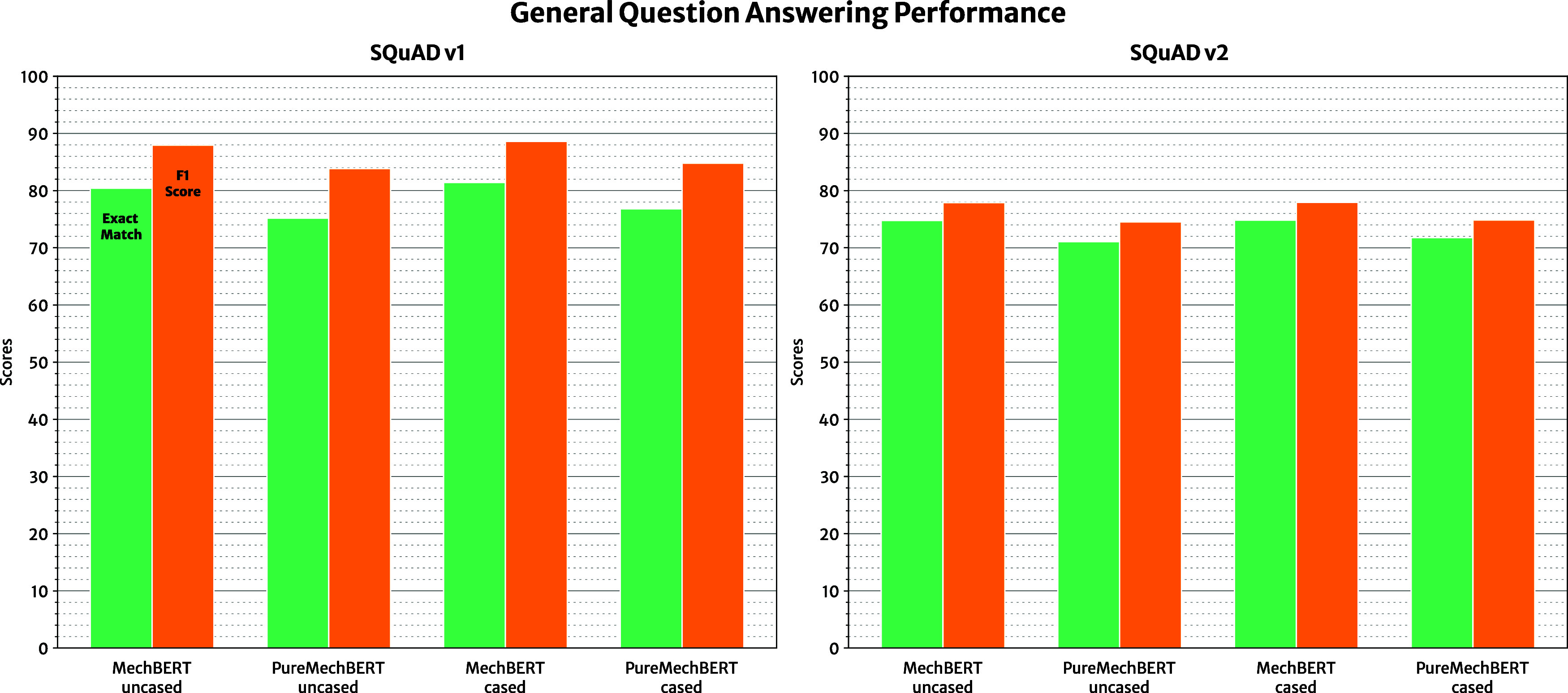
Model performance on general English-language question-answering from the SQuAD data sets. The best performing model on both tasks is the case variant of the MechBERT models.

Overall, the results that evaluate the performance of our language models against general English-language question-answering tasks follow expectations. Across the board, models struggle with data sets containing unanswerable questions due to their added difficulty, and, as such, the exact-match scores are significantly lower for SQuAD v2 which is demonstrated in Figure 9. Extending the pretraining of a BERT model does improve the model's performance, with MechBERT performing slightly better than BERT_BASE_ models. However, the overall improvements are minimal, which is, in part, due to the relatively small pretraining corpus used in this work. PureMechBERT models perform worse for general English-language question-answering tasks.

However, to properly assess the benefit of the pretraining of the BERT models developed in this study, evaluation on a domain-specific question-answering set is required, as is discussed in the following section.

#### Evaluating Domain-Specific Question-Answering Performance

**Figure 10:**
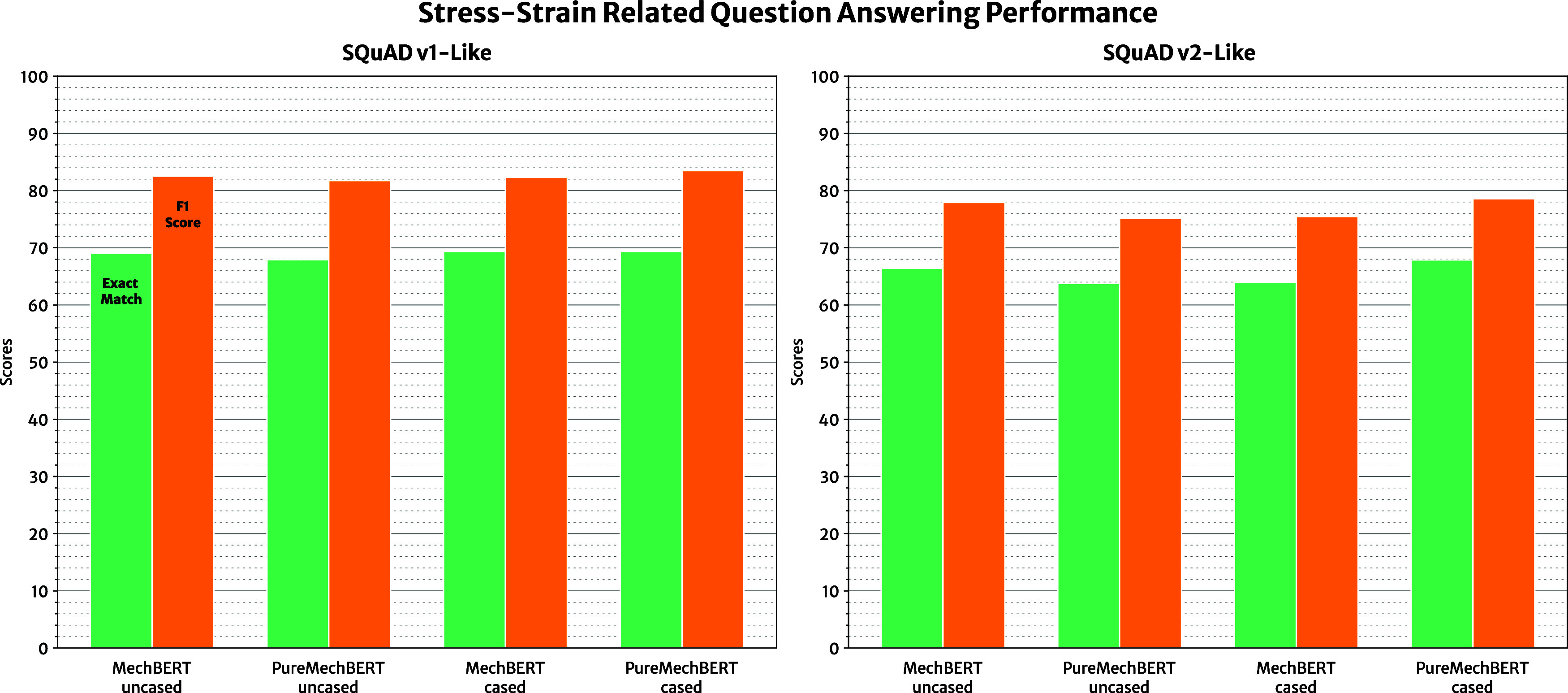
Exact-match and F1 scores on the domain-specific data sets that contain 411 questions related to stress–strain information. The “*SQuAD v1-Like*” results were derived from models fine-tuned on SQuAD v1 and were only evaluated on the answerable questions in the data set. The “*SQuAD v2-Like*” results were from models fine-tuned on SQuAD v2 that were evaluated for all questions, including unanswerable ones.

Our domain-specific data set consists of questions that are more relevant to stress–strain information and, as such, are better equipped to evaluate the ability of our models to perform extractive tasks in the scientific domain. Table 6 provides a summary of the evaluation metrics for each model, which are visualized in Figure 10. The metrics of all models that were tested can be found in the Supporting Information. Note that, while the exact-match score may seem to be relatively low, this is a result of the question-answering data set containing only a single variation of an answer, as opposed to the multiple variations of answers that are contained in the general English-language SQuAD data sets described previously. Therefore, the F1 score gives a more complete description of model performance when assessing domain-specific question-answering on our evaluation data set.

**Table 6:** Performance of Each Fine-Tuned Model on the Domain-Specific Question-Answering Dataset^a^

model version	model	exact	F1
SQuAD v1	MechBERT Cased	69.36	82.32
	MechBERT Uncased	69.08	82.51
	PureMechBERT Cased	69.36	83.50
	PureMechBERT Uncased	67.92	81.75
	BERT_BASE_ Cased	40.75	60.91
	BERT_BASE_ Uncased	43.06	63.09
SQuAD v2	MechBERT Cased	63.99	75.46
	MechBERT Uncased	66.42	77.92
	PureMechBERT Cased	67.88	78.58
	PureMechBERT Uncased	63.75	75.12
	BERT_BASE_ Cased	40.15	53.19
	BERT_BASE_ Uncased	46.72	58.67

a The models fine-tuned on SQuAD v1 are evaluated only on the answerable questions in the dataset. Models fine-tuned on SQuAD v2 are evaluated on the entire dataset.

It is clear that the domain-specific pretraining of a BERT model massively improves its performance when given domain-specific question–answer tasks, with all MechBERT variations performing over 20% better than their BERT_BASE_ counterparts on our question-answering data set. Pretraining on the scientific text that describes stress-related properties embeds more useful knowledge into the language model, which significantly improves performance on downstream tasks within the target domain. This itself is an expected result; however, it is noteworthy that the PureMechBERT models maintain a similar level of proficiency to the MechBERT models, given that they are pretrained on a fraction of the data. In fact, PureMechBERT cased models, fine-tuned on both SQuAD v1 and v2, perform best when they are subjected to domain-specific question-answering tasks with F1 scores of 83.50 and 78.78%, respectively. As such, the relevancy of the pretraining corpus to the target domain of downstream tasks may hold greater importance than purely the amount and variety of pretraining data.

**Figure 11:**
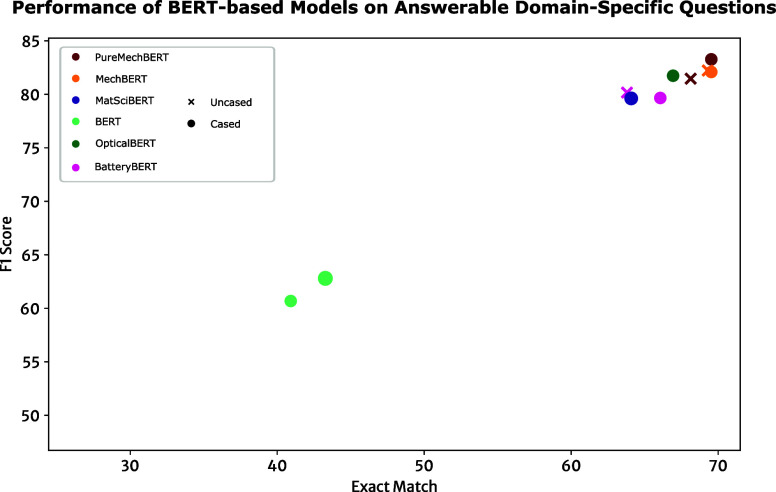
Scatter plot depicting the domain-specific question-answering performance metrics of various cased and uncased BERT models fine-tuned on question-answering tasks from the SQuAD v1 data set. MechBERT includes all models created in this study, the BatteryBERT pretrained on only text pertaining to Battery by Huang et al.,^27^ OpticalBERT includes models trained by Zhao et al.,^28^ BERT represents the original base models by Devlin et al.^17^ and MatSciBERT from Gupta et al.^35^

To determine whether the performance improvements of our language models are due to the corpus being generally scientific or if the topic specificity is of more importance, scientifically aligned BERT models were evaluated on the domain-specific question-answering data set. For this, we performed an evaluation on our models, once fine-tuned on question-answering tasks from SQuAD v1; this is so they could be compared directly to the best performing models found by Huang et al.^27^ and Zhao et al.^28^ which are specialized for the scientific domains of batteries and optical materials, respectively. Also included in our evaluation was the MatSciBERT^35^ model, which was pretrained on scientific language from the overarching domain of material science. A categorized scatter plot of the resulting performance metrics on the domain-specific evaluation data set is illustrated in Figure 11. Overall, BERT models that were pretrained on more scientific data performed better than the BERT_BASE_ models that were pretrained on only the general English language. With increased domain specificity of the pretraining corpus, MechBERT models were able to outperform other scientific BERT models; for example, exact-match scores for PureMechBERT and MechBERT also afford the highest F1 scores, as shown in Figure 11. This result further highlights the impact of specialized pretraining, which can significantly improve the performance of extractive tasks within the domain of interest. This is particularly useful when incorporating language models into information-extraction systems, where the encountered context will be specific to the target domain; as such, a model with a depth of knowledge regarding language patterns in a particular domain is preferred.

#### Evaluating the Influence of the Size and Type of BERT Architecture on Domain-Specific Question-Answering Performance

**Figure 12:**
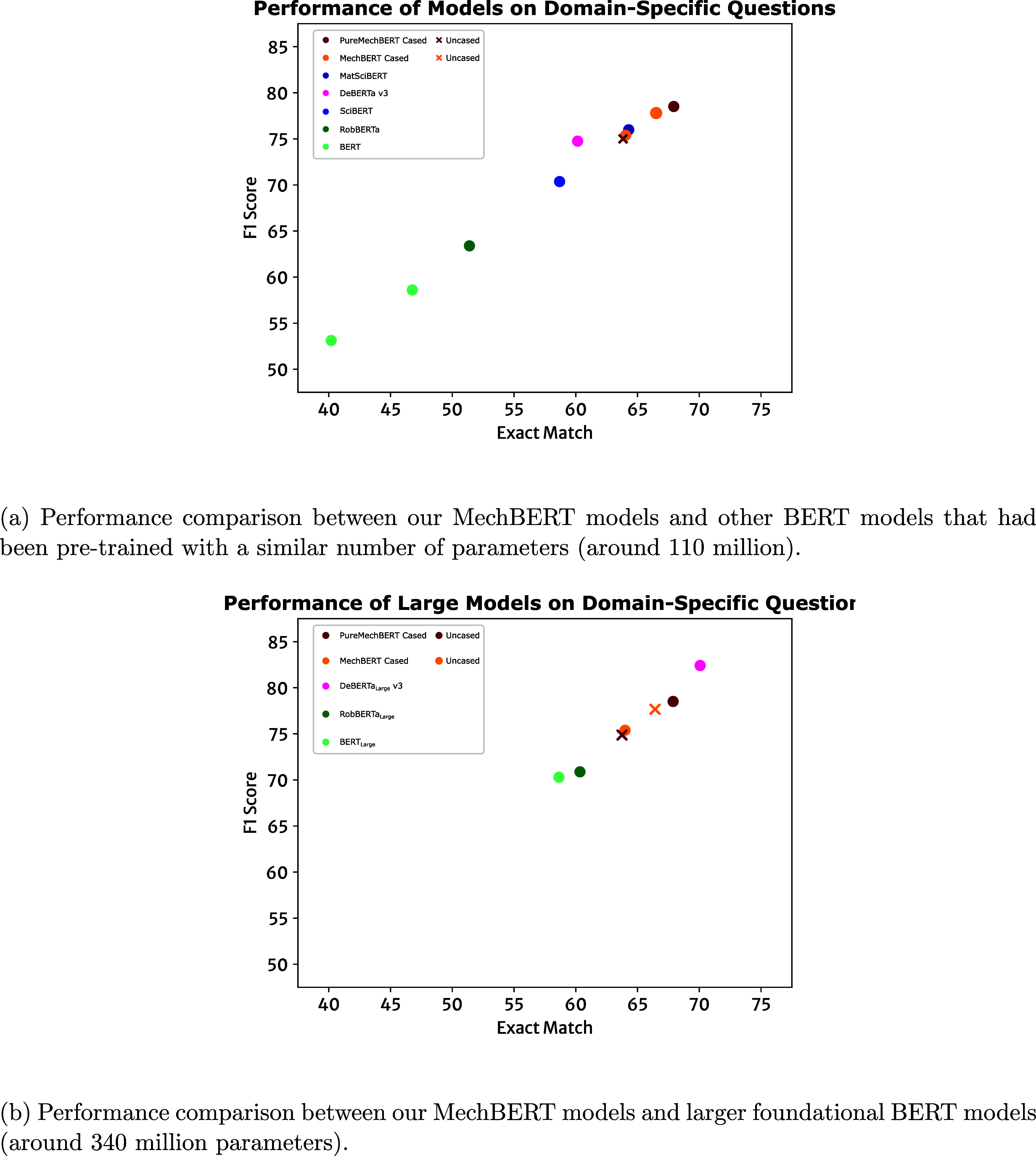
Performance metrics of various BERT-based models when evaluated on domain-specific question-answering tasks. MatSciBERT,^35^ DeBERTa v3,^45^ BERT,^17^ RoBERTa,^20^ and SciBERT^24^ have approximately the same model size as our MechBERT models. DeBERTa_LARGE_ v3, RoBERTa_LARGE_, and BERT_LARGE_ are three times larger than our MechBERT model architecture.

During the course of this study, improvements to the BERT architecture were being developed, owing to the fast-moving nature of the field. Larger foundational BERT models that had been pretrained with many more data and with modified architectures were showcasing state-of-the-art results in question-answering tasks. To this end, the SQuAD v2 variants of the fine-tuned MechBERT models were compared to updated foundational language models that are primarily based on the BERT architecture, such as RoBERTa^20^ and DeBERTa,^44,45^ using fine-tuned versions of these models produced by the Deepset team.^43^Figure 12a presents a performance comparison of BERT models that had been pretrained with a similar number of parameters employed in this study (approximately 110 million). Figure 12b presents the performance levels of BERT models that had been pretrained with many more parameters (exceeding 340 million parameters) relative to that of our MechBERT models.

Figure 11a shows that the newer foundational BERT-model architectures demonstrate enhanced performance over the original BERT models, with F1 scores of 63.46 and 74.82% being achieved on the domain-specific evaluation set for RoBERTa and DeBERTa v3, respectively. Indeed, the up-to-date foundational BERT architecture and improved pretraining objectives employed in the general English-language model, DeBERTa v3^45^ even outperforms SciBERT which was pretrained on the scientific literature and is thus theoretically more suited to domain-specific question-answering tasks. The MatSciBERT model, with a pretraining corpus that is closely related to MechBERT models, also demonstrates good performance. Nonetheless, MechBERT models maintain the best performance, exceeding the next best model by 7.78% in an exact-match score and 3.76% in the F1 score. This superior performance is due to the specialized pretraining corpus and reinforces our conclusions made above. To reiterate, the domain-specificity of the corpus is crucial for the performance of BERT-based language models in downstream tasks within the target domain, as opposed to the size of the pretraining corpus alone.

Figure 12b presents the level of performance of BERT models that had been pretrained on many more parameters (exceeding 340 million) relative to that of our MechBERT models. There is a prevailing assumption that larger foundational language models are more capable, given that they have been constructed using many more parameters and have been pretrained on a far more extensive corpus (of the order of 160 GB) and that they should therefore perform better on question-answering tasks than smaller language models. For example, the large variants, RoBERTa_LARGE_ and DeBERTa_LARGE_ v3, achieve significantly improved F1 scores of 84.03 and 90.75% when fine-tuned with question-answering tasks from the general English-language SQuAD v2 data sets, respectively.^20,45^ Notably, all MechBERT language models are at least three times smaller in terms of the number of parameters, and PureMechBERT models were pretrained with only 7.4 GB of text data. Nonetheless, all variants of our MechBERT models can compete with these larger BERT-based models. For example, the PureMechBERT cased model outperforms RoBERTa_LARGE_ and BERT_LARGE_ on domain-specific question-answering by 7.63 and 8.22% in F1 score, and 7.54 and 9.24% in exact-match score, respectively. In doing so, the smaller models are also able to process the samples at six times the speed of the larger models, making them more appealing for an information-extraction system that needs to balance precision with performance. This finding is also important from an energy sustainability perspective; smaller language models could offer a better economy through more modest energy consumption.

The only foundational language model that was deemed to be superior to the ones developed in this study is DeBERTa_LARGE_ v3, as illustrated in Figure 12b. This is a direct result of the introduction of disentangled attention and a modified pretraining objective in the development of DeBERTa_LARGE_ v3. In the original BERT models, a standard self-attention mechanism is used, and the pretraining objective is masked language modeling. In contrast, DeBERTa v3 disentangles this attention mechanism into two, one for word content and one for position, and uses the Replaced Token Detection pretraining objective.^45^ These architectural differences result in improved model performance for downstream tasks and present a potential avenue for further improvements for our MechBERT models. Nevertheless, the difference between PureMechBERT and DeBERTa v3 (2.19% in exact-match score and 3.9% in F1 score) is smaller than the difference between the MechBERT models and other large BERT-based models. Our models outperform BERT-based models of the same size and maintain relevancy when compared to larger BERT models for use in downstream tasks within stress–strain-related domains. We have therefore demonstrated that our MechBERT models are powerful tools within the stress-engineering materials domain for extractive use cases. Moreover, they can compete with other state-of-the-art language models while being smaller in size, enabling faster processing and requiring a relatively small fraction of data to pretrain them. Our small language models therefore stand to offer a “win–win” formula of greater operational efficiency by more energy-sustainable means.

### BERT Viz

**Figure 13:**
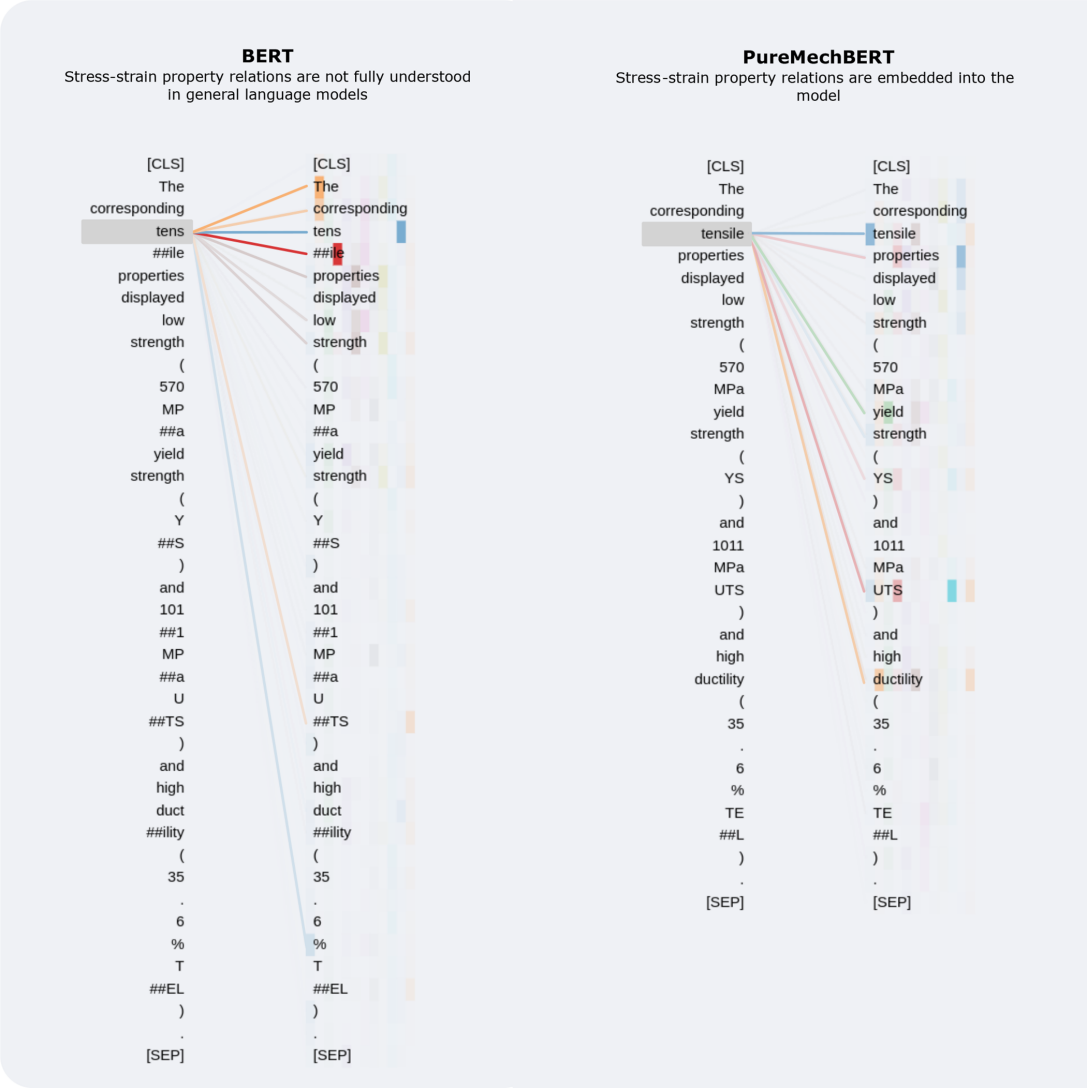
Visualization of attention^46^ for BERT_BASE_ and PureMechBERT models on the example sentence: “The corresponding tensile properties displayed low strength (570 MPa yield strength (YS) and 1011 MPa UTS) and high ductility (35.6% TEL)”.

The BERT architecture contains multiple attention heads that are used to process different aspects of a text sequence and learn the interrelations between words. A visualization of these attention patterns provides an intuitive view of how a language model views linguistic patterns and relationships. The attention patterns produced by the 12 attention heads in BERT and PureMechBERT models were obtained using the BertViz software^46^ on an example sentence sourced from the materials-engineering literature. For instance, Figure 13 portrays a side-by-side view of how the BERT and PureMechBERT models relate the word *“tensile”* to other tokens in the sentence and provides a visual comparison of the understanding of scientific terminology by each model. The self-attention in this visualization is represented by a line connecting each token in the sequence, with a different color being used to distinguish the different attention heads. The weight of the connection reflects the attention score; more opaque colors represent stronger relations between the respective tokens. For the BERT_BASE_ model, which was primarily pretrained on general English-language text, the relation of the word “*tensile*” is mostly focused on adjacent words, with no significant connections to mentions of material properties. In stark contrast, the attention heads in the PureMechBERT models display a high attention score between “*tensile*” and mentions of tensile properties, such as “*UTS*”, “*YS*”, and “*ductility*”.

As such, there is evidence that domain-specific pretraining allows the PureMechBERT models to capture the unique contextual relations between stress–strain properties which a general purpose language model cannot see. Qualitatively, this suggests that the PureMechBERT models, and MechBERT models to a lesser extent, have embedded knowledge of tensile properties and their representations within text that can be utilized for downstream tasks; from this, we presume that the same is true of other stress–strain properties. Moreover, it is worth remembering that these models have been learned from unlabeled text automatically, without the laborious task of manually designing such complex representations, thereby overcoming the limitations present in conventional information-extraction systems.

## Conclusions

In summary, four language models based on the BERT architecture have been pretrained using text sourced from the stress–strain-related scientific literature: MechBERT cased and uncased models were initialized from the original BERT weights, while PureMechBERT cased and uncased models were initialized from scratch. These models were fine-tuned for general English-language question-answering tasks using the SQuAD v1 and v2 data sets, resulting in eight fine-tuned models ready for extractive use cases. Bayesian optimization was employed to discover the best hyperparameter combination for fine-tuning, which proved to be useful as the exact-match score could deviate by 6.1% on average depending on the model configuration. Evaluation was conducted using the general English-language SQuAD data sets, and it was found that the further pretraining did not negatively affect the performance of MechBERT models and, in most cases, offered improvements when compared to the original BERT models.

An additional evaluation set was constructed using an annotation tool that was custom-built for this work which followed the guidelines of the SQuAD data sets. This contained questions and answers that specifically relate to stress–strain properties, with the context being sourced from articles that are not in the pretraining corpus; as such, the data set is better equipped to evaluate the performance of the language models on domain-specific downstream tasks.

It was found that the MechBERT models outperform others on question-answering tasks within the materials-engineering domain. The best-performing model, for both SQuAD v1 and v2 versions, was the PureMechBERT cased variant. This outperformed other models, even those that were larger and pretrained on more data, demonstrating the significant benefit of focused pretraining for use cases within a specific field. To the best of our knowledge, these models are the first Transformer-based language models that are specialized in stress–strain information and showcase elevated performance for in-domain extractive tasks.

This paper has exemplified an alternative approach to information extraction in domains of research that rely on specialist vernacular; this approach overcomes the problems faced with conventional NLP-based methods through the use of domain-specific language models. The variants of our MechBERT models automatically learn the linguistic patterns of scientific text. Meanwhile, an understanding of stress–strain property semantic relations is embedded into the model, owing to the specificity of the pretraining corpus; a task that is complex and difficult to approach manually. A visualization of the attention patterns of MechBERT models provides evidence that some understanding of tensile properties and their semantic relation to one another is embedded during the pretraining stages of the language models. The same level of understanding is not present in general English-language BERT models. As such, it is reasonable to assume that the domain-specific knowledge representations have automatically been learned and can be utilized for not only information-extraction purposes but also other downstream NLP tasks.

The extractive capabilities of these property-specific language models have been showcased; it has been demonstrated that MechBERT variants outperform related models on question-answering tasks surrounding stress–strain information, with an increase of 25.39% (answerable questions) and 22.59% (unanswerable questions included) in the F1 score over BERT_BASE_ counterparts. Due to the nature by which extractive questions target information about materials and their associated properties, the improvements in performance highlight that the stages which one would normally associate with a conventional information-extraction pipeline have been properly learnt by the language model, and they can therefore be successfully implemented for the purpose of information extraction. While further testing is required to determine the efficacy of these language models in a fully fledged information-extraction system, initial results show that MechBERT models are capable of achieving an F1 score of up to 83.50% in extractive tasks within the domain.

As a secondary observation, the significant impact of specialized pretraining for downstream performance within that domain has been realized. PureMechBERT models have been found to outperform other language models on the domain-specific evaluation set, even those that have more pretraining data that are still closely related, such as MatSciBERT,^35^ SciBERT,^24^ and other scientifically aligned BERT models. PureMechBERT impressively outperformed larger BERT-based language models, such as BERT_LARGE_ and RoBERTa_LARGE_, in domain-specific question-answering tasks. These large variants are three times the size of our MechBERT models, in terms of model parameters and have been pretrained on many more data; for example, the RoBERTa and DeBERTa v3 models have been pretrained on a total of 160 GB of text data.^20,45^ In comparison, the PureMechBERT models have been pretrained on only a 7.4 GB corpus, i.e., less than 5% of the larger models; yet they are still able to achieve better results on the domain-specific data set by 7.63 and 7.54% in F1 score and exact-match score, respectively, when compared to RoBERTa_LARGE_. Our PureMechBERT model is able to outperform the DeBERTa v3 model of the same size; although, it is beaten by the large DeBERTa v3 variant. Despite this, the disparity between PureMechBERT and DeBERTa_LARGE_ v3 models (3.9% F1 score and 2.19% exact-match score) is smaller than that between the PureMechBERT model and other large BERT models. Moreover, since PureMechBERT is a much smaller model than DeBERTa_LARGE_ v3, it is able to process samples at a rate six times faster. This is valuable for information-extraction systems, which need to balance precision with performance. This finding has pertinent implications for language-model applications from a perspective of improving the operational efficiency of AI-based processes while simultaneously gaining energy sustainability.

## Data Availability

All of the scripts used to pretrain the MechBERT and PureMechBERT models are available online, as is the code that was used to process and evaluate the models.^48^ The QAannotation tool used to create the domain-specific evaluation data set is available online.^42^ The evaluation data set itself is provided in Supporting Information. The pretrained and fine-tuned models are available on the Molecular Engineering Group HuggingFace.^49^

## Supporting Information Available

Details on pretraining hyperparameters, fine-tuning hyperparameter optimization, domain-specific evaluation summary for all models tested; document object identifiers (DOIs) for each paper that was used as the input corpus that trained the MechBERT models (partitioned into three DOI lists that pertain to papers from three publishing houses); and discussion and comparison between ChatGPT and our MechBERT models in the context of extractive question-answering for research studies (PDF)
