# Supplementary material for: MechBERT: Language Models for Extracting Chemical and Property Relationships about Mechanical Stress and Strain
Source: J Chem Inf Model. 2025 Jan 31;65(4):1873–88. doi: 10.1021/acs.jcim.4c00857 (PMC11863389; doi:10.1021/acs.jcim.4c00857)
Supplement: Supplementary file 1 — ci4c00857_si_001.pdf [file ci4c00857_si_001.pdf]

Supporting Information for: MechBERT: Language Models for Extracting Chemical and Property Relationships about Mechanical Stress and Strain

Pankaj Kumar,[†,‡,¶] Saurabh Kabra,[‡,§] and Jacqueline M. Cole[*,†,‡,¶]

†*Cavendish Laboratory, Department of Physics, University of Cambridge, J. J. Thomson Avenue, Cambridge, CB3 0HE. U.K.*

‡*ISIS Neutron and Muon Source, STFC Rutherford Appleton Laboratory, Harwell Science and Innovation Campus, Didcot, OX11 0QX, U.K.*

¶*Research Complex at Harwell, Rutherford Appleton Laboratory, Harwell Science and Innovation Campus, Didcot, Oxfordshire OX11 0FA, U.K.*

§*Current address: Neutron Sciences Directorate, One Bethel Valley Rd, Oak Ridge, TN 37831, United States*

E-mail: jmc61@cam.ac.uk

Phone: +44 (0)1223 337470

Supporting Information Available

Pre-training Details

In this study, multiple variations of models were tested. To avoid exhausting the available computing resources, only single versions (either cased or uncased) were used for experimentation. Firstly, the batch size and pre-training steps were assessed by pre-training a model without the adjustments to optimize the speed of pre-training. The *cased-batch256* model represents a cased model pre-trained with a batch size of 256 and for 1 million total steps. Also, some other studies employed a learning rate of 1×10^{-4}, to test if this would have a significant impact on the final results, an uncased model was pre-trained with this learning rate (*LR1-uncased*). As shown in Table 1, the increased batch size and reduced pre-training steps had no significant effect on the performance of the final fine-tuned models. Therefore, the pre-training in this study is more efficient as it is conducted in much less time while maintaining performance. Similarly, the initial learning rate did not meaningfully influence the final performance.

Table 1: Summary of all fine-tuned models, including the optimal hyperparameter configuration and the resulting exact-match score.

**Model Version**	**Model**	**Learning Rate**	**Epochs**	**Batch Size**	**Exact Match (%)**
SQuAD v1	cased-batch256	3.85962516931e-05	4	6	80.70
	LR1-uncased	9.08697427712e-05	3	33	80.35
	PureMechBERT Cased	2.34149250752e-05	4	6	76.81
	PureMechBERT Uncased	6.65858503326e-05	3	35	75.18
	MechBERT Cased	5.75409064017e-05	3	15	81.41
	MechBERT Uncased	6.69796562345e-05	3	11	80.41
SQuAD v2	LR1-uncased-sq2	8.25973756347e-05	2	12	74.87
	PureMechBERT Cased	4.90973747641e-05	3	19	71.77
	PureMechBERT Uncased	3.80820446232e-05	4	15	71.06
	MechBERT Cased	6.40600319349e-05	2	32	74.84
	MechBERT Uncased	0.0001494572498569	2	19	74.78

Fine-tuning Hyperparameter Optimization

Exploiting the computationally inexpensive nature of fine-tuning, over 100 configurations for each model, for both SQuAD v1 and v2 tasks, were generated. Iteratively, each configuration improved on the last using Bayesian optimization techniques provided by the Weights and Bias framework. As a demonstration of the benefit of using this method, Table 2 details the standard deviation of exact match and F1 score calculated over the optimization process, showing how vastly different the final performance of the fine-tuned models can be.

Table 2: Standard deviation of performance metrics calculated during the optimization of fine-tuning hyperparameters.

**Model Version**	**Name**	**Exact Match**	**F1**
SQuAD v1	cased-seq256	16.925	18.124
	LR1-uncased	1.528	1.168
	PureMechBERT Cased	4.818	4.418
	PureMechBERT Uncased	2.928	2.541
	MechBERT Cased	6.983	6.834
	MechBERT Uncased	5.704	5.039
SQuAD v2	LR1-uncased-sq2	3.431	3.451
	PureMechBERT Cased	2.418	2.589
	PureMechBERT Uncased	2.448	2.592
	MechBERT Cased	5.674	5.935
	MechBERT Uncased	18.398	19.295

Figures 1 to 3 summarize the best 100 configurations for each model, with the best configuration highlighted in red. The best configurations are also detailed above in Table 1.

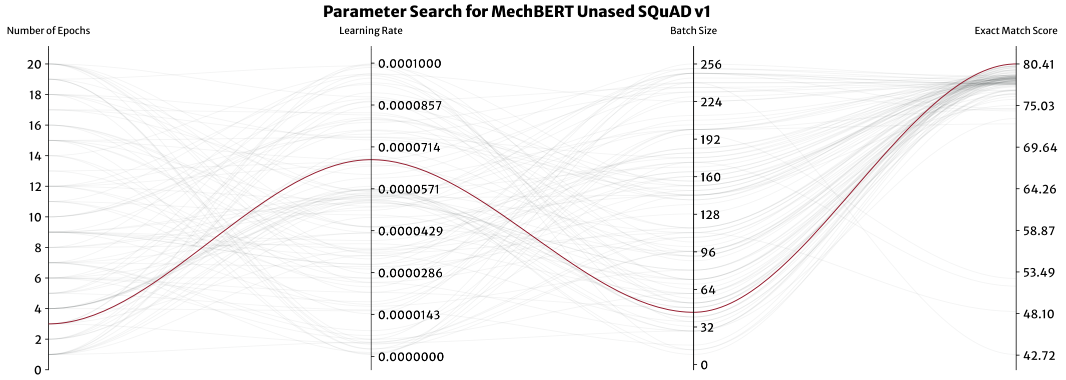

(a) SQuAD v1

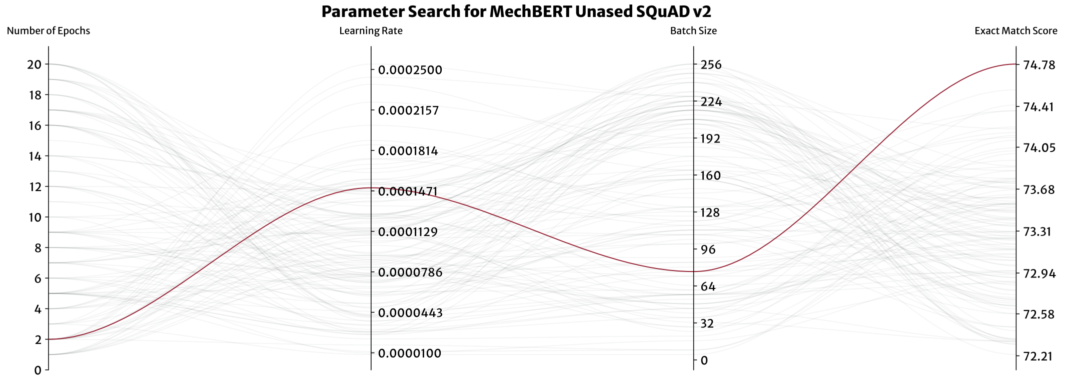

(b) SQuAD v2

Figure 1: Summary of the top 100 hyperparameters found during the optimization of fine-tuned *MechBERT* uncased models. All configurations are compared using the exact-match score and the best performing model is highlighted in red.

Domain-specific evaluation summary

To compare the MechBERT models against other related works, the best fine-tuned models were sourced and tested against the domain-specific question answering set. A summary of the metrics found is detailed in Table 3. Models were only compared if they were available, for example, related work such as OpticalBERT[1] and BatteryBERT[2] only fine-tuned their models on SQuAD v1 and as such, are not tested on the unanswerable questions in this study.

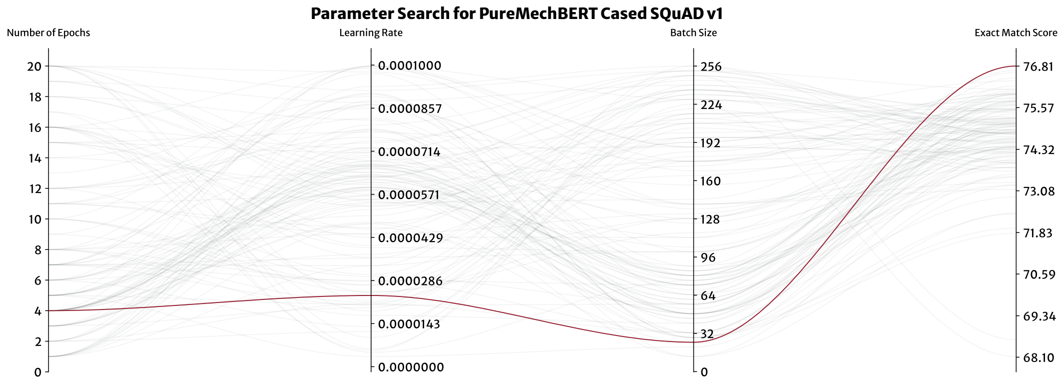

(a) SQuAD v1

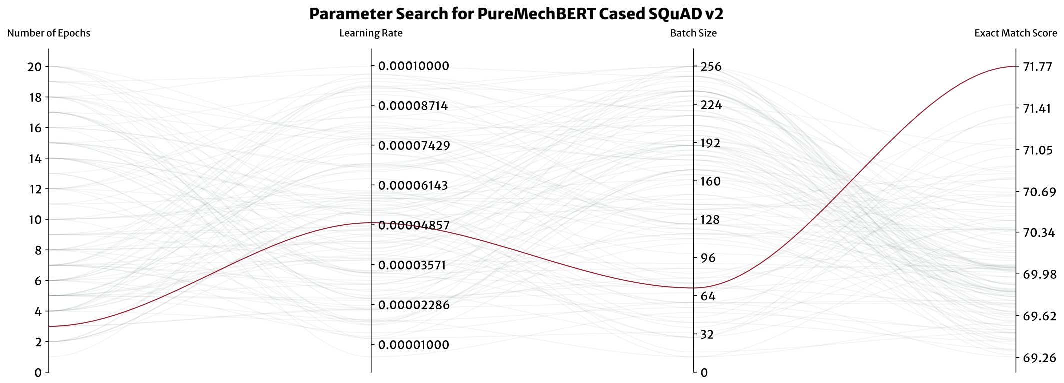

(b) SQuAD v2

Figure 2: Summary of the top 100 hyperparameters found during the optimization of fine-tuned *PureMechBERT* cased models. All configurations are compared using the exact-match score and the best performing model is highlighted in red.

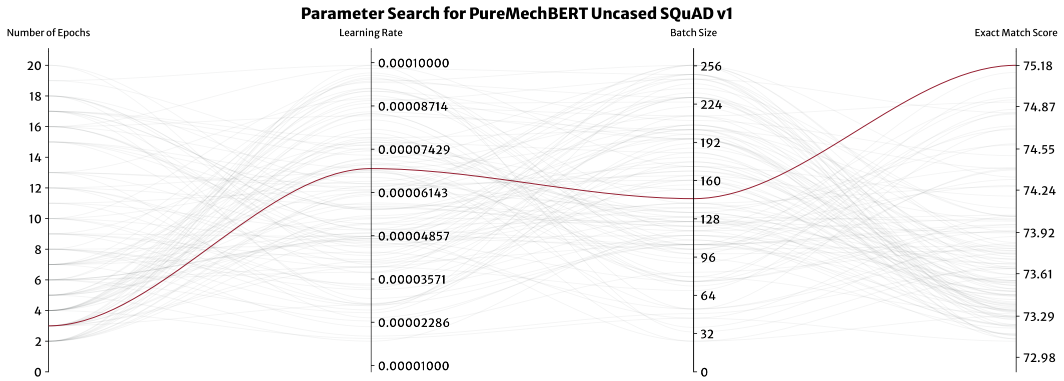

(a) SQuAD v1

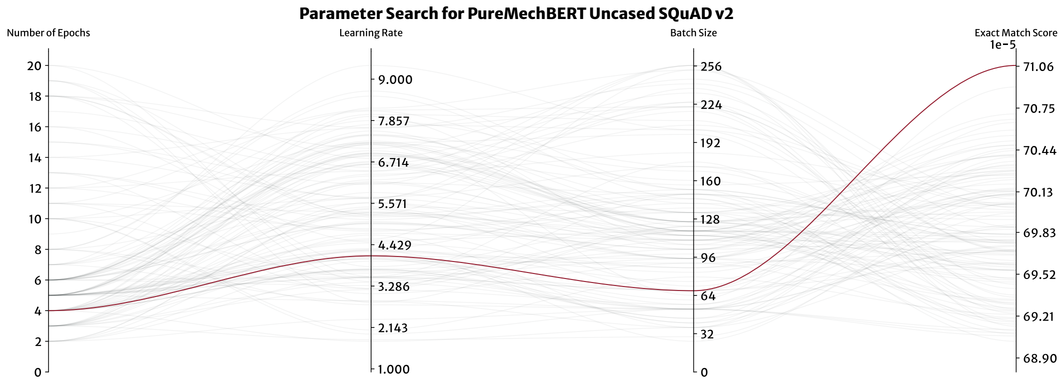

(b) SQuAD v2

Figure 3: Summary of the top 100 hyperparameters found during the optimization of fine-tuned *PureMechBERT* uncased models. All configurations are compared using the exact-match score and the best performing model is highlighted in red.

Table 3: Full list of evaluation metrics of all tested models on domain-specific question answering.

Variant	Model Name	Exact	F1
SQuAD v1	PureMechBERT cased	69.36	83.50
	MechBERT cased	69.36	82.32
	MechBERT uncased	69.08	82.51
	PureMechBERT uncased	67.92	81.75
	BERTbase cased	40.75	60.91
	BERTbase uncased	43.06	63.09
	BatterySciBERT uncased	66.76	81.52
	BatterySciBERT cased	26.59	48.47
	BatteryonlyBERT uncased	63.58	80.45
	BatteryonlyBERT cased	65.90	79.89
	OpticalPureBERT	67.20	81.76
	OpticalBERT	65.76	80.94
	MatSciBERT	63.87	79.91
SQuAD v2	PureMechBERT cased	67.88	78.58
	MechBERT cased	63.99	75.46
	MechBERT uncased	66.42	77.92
	PureMechBERT uncased	63.75	75.12
	BERTbase cased	40.15	53.19
	BERTbase uncased	46.72	58.67
	RoBERTa	51.34	63.46
	SciBERT	58.64	70.43
	DeBERTa v3	60.10	74.82
	DeBERTa v3 Large	70.07	82.48
	RoBERTa Large	60.34	70.95
	BERT Large	58.64	70.36

Comparison to ChatGPT

We have compared our MechBERT models against those that employ a similar model architecture. The primary focus in this study has been on bidirectional encoder based model architectures as they are inherently more suited for extractive and other language tasks requiring exact and precise data retrieval. While generative pre-trained transformer-based language models, such as those developed by OpenAI, are indeed powerful tools for natural language tasks and achieve competitive performance on numerous NLP benchmarks, they tend to underperform in discriminative tasks including exact question-answering from a target context.[3,4] For example, on the SQuAD 2.0 benchmark, ChatGPT performs worse than the current state-of-the-art and the BERT models tested in this study; Laskar *et al.* reported that ChatGPT achieves a score of 66.9% on SQuAD 2.0, where the answers are assessed via lexical or fuzzy word matching.[5] The performance is often hindered because ChatGPT may "hallucinate" new information or introduce information that is not present in the provided context.

To illustrate, Figure 4 shows that, when directly supplying ChatGPT with a context paragraph and an extractive question, the model produces a generated answer that is more conversational, rather than returning the exact tokens sought within the text. Techniques such as few-shot prompting or more detailed instructions can be employed in an attempt to guide the model to generate the answers that we seek, as demonstrated in Figure 4. Regardless, ChatGPT primarily functions as a conversational agent as it was intended; fine-tuning a GPT model for a different task would require access to the proprietary API which is constrained and limits flexibility for research projects. The models powering ChatGPT are closed-sourced, with no direct access to the model weights or architecture specifics. This lack of transparency is not conducive for open-research. Our MechBERT models, on the other hand, are entirely open-source, with full disclosure of all training details. This ease of access and lack of restrictions allow researchers to use, modify, and distribute MechBERT models in ways that align with open-source practices and promote more open research.

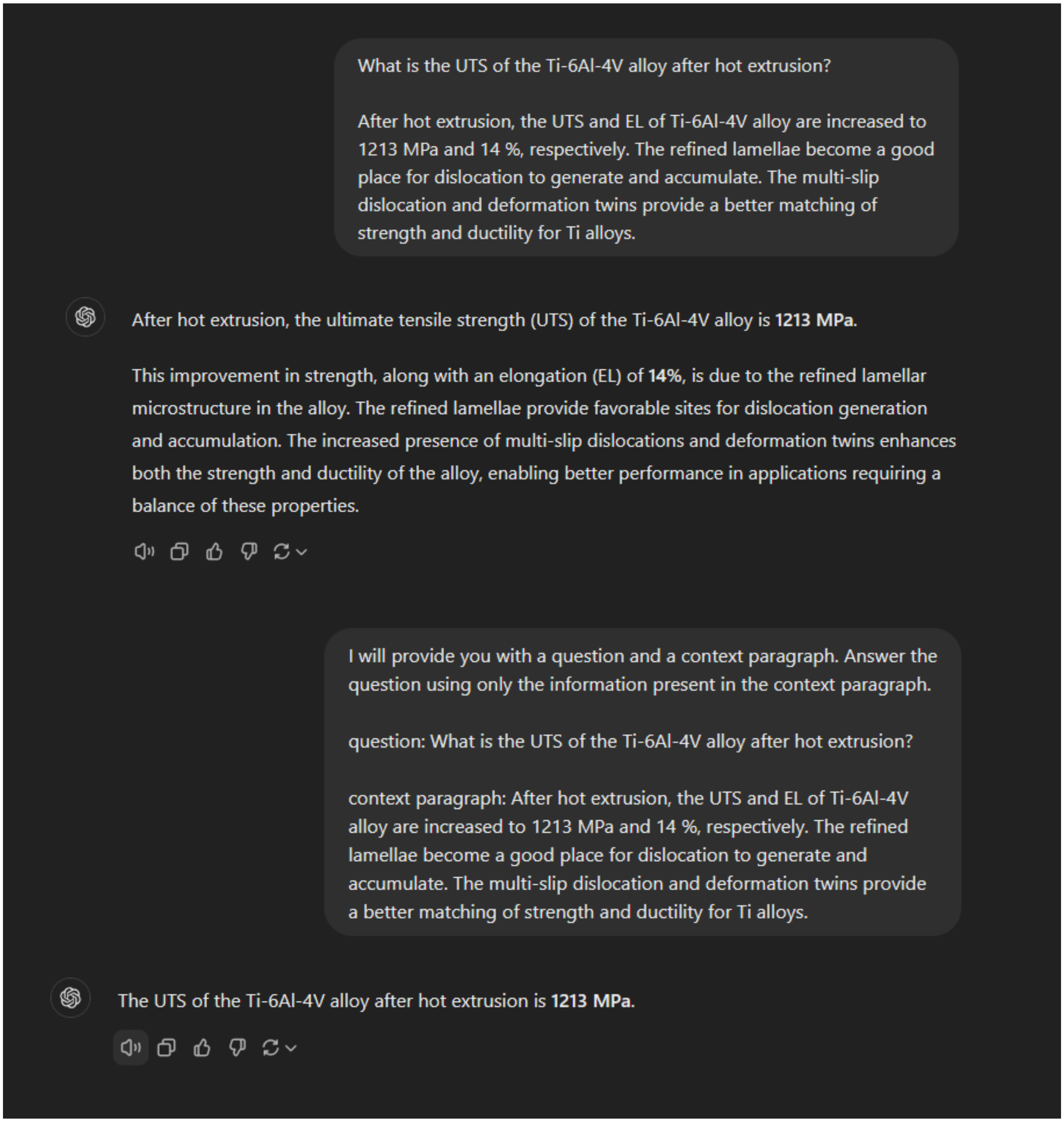

Figure 4: Example prompts instructing ChatGPT to perform extractive question answering. The first prompt does not provide the conversational agent with any instructions resulting in a generated answer that needs further processing to extract the exact value. The second prompt includes more instructions to the agent resulting in a correct answer without unnecessary details.

From a practical standpoint, BERT-based models are generally smaller in terms of model size, thus they are more resource-efficient and require less computational power to fine-tune

or deploy. As mentioned, this aspect is compelling from an energy-sustainability perspective, especially when performance on downstream tasks is not hindered from the use of a smaller model. Our MechBERT models have herein been demonstrated to be competitive with larger models in domain-specific question-answering and thus, other researchers may also find success building upon our MechBERT models for specialised solutions. This is in contrast to ChatGPT, whose larger models require more computational resources for deployment, inference, and fine-tuning in addition to the monetary cost of accessing the closed-source model itself. For applications that do not require generative capabilities, there is little benefit to using GPT-based language models such as ChatGPT, when other, more resource-efficient and equally performing, language models exist.

References

(1) Zhao, J.; Huang, S.; Cole, J. M. OpticalBERT and OpticalTable-SQA: Text- and Table-Based Language Models for the Optical-Materials Domain. *Journal of Chemical Information and Modeling* **2023**, *63*, 1961–1981.

(2) Huang, S.; Cole, J. M. BatteryBERT: A Pretrained Language Model for Battery Database Enhancement. *Journal of Chemical Information and Modeling* **2022**, *62*, 6365–6377.

(3) Bahak, H.; Taheri, F.; Zojaji, Z.; Kazemi, A. Evaluating ChatGPT as a Question Answering System: A Comprehensive Analysis and Comparison with Existing Models. 2023; https://arxiv.org/abs/2312.07592.

(4) Zhong, Q.; Ding, L.; Liu, J.; Du, B.; Tao, D. Can ChatGPT Understand Too? A Comparative Study on ChatGPT and Fine-tuned BERT. 2023; https://arxiv.org/abs/2302.10198.

(5) Laskar, M. T. R.; Bari, M. S.; Rahman, M.; Bhuiyan, M. A. H.; Joty, S.; Huang, J. A

Systematic Study and Comprehensive Evaluation of ChatGPT on Benchmark Datasets. Findings of the Association for Computational Linguistics: ACL 2023. Toronto, Canada, 2023; pp 431–469.
